# A microbiome and metabolomic signature of phases of cutaneous healing identified by profiling sequential acute wounds of human skin: An exploratory study

**DOI:** 10.1371/journal.pone.0229545

**Published:** 2020-02-27

**Authors:** Mohammed Ashrafi, Yun Xu, Howbeer Muhamadali, Iain White, Maxim Wilkinson, Katherine Hollywood, Mohamed Baguneid, Royston Goodacre, Ardeshir Bayat

**Affiliations:** 1 Plastic & Reconstructive Surgery Research, Division of Musculoskeletal & Dermatological Sciences, NIHR Manchester Biomedical Research Centre (BRC), University of Manchester, Manchester, United Kingdom; 2 Manchester University NHS Foundation Trust, Wythenshawe Hospital, Manchester, United Kingdom; 3 Bioengineering Group, School of Materials, University of Manchester, Manchester, United Kingdom; 4 School of Chemistry, Manchester Institute of Biotechnology, The University of Manchester, Manchester, United Kingdom; 5 Department of Biochemistry, Institute of Integrative Biology, University of Liverpool, Liverpool, United Kingdom; 6 Laboratory for Environmental and Life Sciences, University of Nova Gorica, Nova Gorica, Slovenia; 7 Manchester Centre for Synthetic Biology of Fine and Speciality Chemicals (SYNBIOCHEM), Manchester Institute of Biotechnology, The University of Manchester, Manchester, United Kingdom; Laurentian University, CANADA

## Abstract

Profiling skin microbiome and metabolome has been utilised to gain further insight into wound healing processes. The aims of this multi-part temporal study in 11 volunteers were to analytically profile the dynamic wound tissue and headspace metabolome and sequence microbial communities in acute wound healing at days 0, 7, 14, 21 and 28, and to investigate their relationship to wound healing, using non-invasive quantitative devices. Metabolites were obtained using tissue extraction, sorbent and polydimethylsiloxane patches and analysed using GCMS. PCA of wound tissue metabolome clearly separated time points with 10 metabolites of 346 being involved in separation. Analysis of variance-simultaneous component analysis identified a statistical difference between the wound headspace metabolome, sites (*P* = 0.0024) and time points (*P*<0.0001), with 10 out of the 129 metabolites measured involved with this separation between sites and time points. A reciprocal relationship between *Staphylococcus* spp. and *Propionibacterium* spp. was observed at day 21 (*P*<0.05) with a statistical correlation between collagen and *Propionibacterium* (*r* = 0.417; *P* = 0.038) and *Staphylococcus* (*r* = -0.434; *P* = 0.03). Procrustes analysis showed a statistically significant similarity between wound headspace and tissue metabolome with non-invasive wound devices. This exploratory study demonstrates the temporal and dynamic nature of acute wound metabolome and microbiome presenting a novel class of biomarkers that correspond to wound healing, with further confirmatory studies now necessary.

## Introduction

Delayed wound healing occurs as a result of deficiencies in wound healing processes [1,2], resulting in chronic wounds. This can affect between 3–5% of patients over the age of 65 and presents an ever increasing burden on the healthcare system costing upward of $20 billion in the US alone [3,4]. In view of our limited understanding of the mechanisms that inhibit cutaneous wound healing in humans, various “omic” technologies have emerged in recent years, such as genomics, proteomics, transcriptomics and metagenomics. These have been utilised and developed to provide a deeper understanding of the pathways responsible for normal and impaired wound healing as well as help identify some of the biomarkers involved [5].

Metabolomics is a multidisciplinary science that seeks to define the entire complement of metabolites (low molecular weight organic and inorganic chemicals) within a biological system of interest [6]. This opens up the possibilities of identifying novel biomarkers. As an emerging field, it has several advantages over other “omic” technologies as it provides the final downstream products of transcription and translation [7]. Its highly dynamic nature allows it to provide the closest links to the system phenotype as well as it is a real time measure [8].

Our knowledge of the metabolic profile of wounds and their role in the wound healing processes is limited. The metabolome of wound healing has been understudied. Only two animal studies [9,10] and three human studies [11–13] have investigated the metabolic profile of cutaneous wounds at a single time-point using a metabolomics approach. In addition other “omic” technologies such as genomics have been utilised to assess the progressive changes in gene expression in wound healing over time [14,15]. However, the temporal variations in the wound metabolome in normal wound healing are yet to be investigated.

Whilst persistent wound infection is a major contributor to delayed wound healing [16], the dynamic nature of the cutaneous microbiome plays a key role in resolving acute, non-resolving acute and chronic wounds [17,18]. The wound microbiome can have a detrimental effect on wound healing but by contrast may also positively influence successful healing [19,20]. Therefore, understanding the relationship between different microbial communities in the wound and the eventual outcome of wound healing is of great importance, as there is scope to manipulate the microbiome in diseased states using targeted therapy. With the advent of DNA sequencing techniques, our ability to accurately characterise the microbiome has been revolutionised. However, our understanding of how the cutaneous microbiome dynamically alters during the normal wound healing phases is limited.

Therefore, the aims of this unique exploratory sequential temporal acute cutaneous wound healing study in humans were: To characterise the dynamic wound metabolome and the wound microbiome in normal acute wound healing; and secondly, to investigate their relationship to acute wound healing processes. In order to achieve these aims, the study was conducted in two parts. Part one involved the detection of wound surface metabolites (wound headspace metabolome) using sorbent material (Tenax/Unicarb, Markes’ International, Llantrisant, UK). It also involved the processing of the wound tissue for microbiome analysis through metagenomic sequencing. Part two again involved the detection of the wound headspace metabolome, however, using polydimethylsiloxane (PDMS (Goodfellow Cambridge Ltd., Huntingdon, UK)), as an alternate sorbent material in an attempt to improve the detection of metabolites. The wound tissue in part 2 of the study was processed for metabolite extraction and detection from the wound tissue (wound tissue metabolome). During both parts of the study various parameters associated to wound healing processes were objectively measured using non-invasive imaging modalities.

## Materials and methods

### Study outline

A temporal sequential design punch biopsy multi-part study was conducted to determine the metabolomic and microbiome profiles of acute wound healing (S1 Fig). This study was conducted in accordance with the ethical principles of Good Clinical Practice and the Declaration of Helsinki Principles.

### Part 1

#### Study participants

Healthy participants were enrolled into the study at the Manchester University NHS Foundation Trust, England, UK between February and March 2017. The University of Manchester research ethics committee and Manchester University NHS Foundation Trust research and development department approval were granted for the study (Ethics number: 16045). All participants deemed suitable for enrolment (inclusion and exclusion criteria outlined in S1 Table), provided written informed consent. Five participants were recruited (3 Male), with an age range between 21–51 years.

#### Study design

On day 0, participants had two 4 mm diameter full thickness skin biopsies, performed under local anaesthetic (1% Lidocaine), in each of their upper inner arms leading to the creation of four iatrogenic wounds. Full thickness skin biopsy was defined as removing the entire epidermis and dermis to expose hypodermic fat. The punch biopsies were performed 5 cm from the axillary hairline and parallel to the medial epicondyle and were 3 cm in distance from each other. Pressure was applied to each biopsy site until haemostasis had been achieved. The biopsies were processed for microbial profiling as detailed below. Full thickness skin/wound biopsies were taken for microbiological analysis as superficial skin sampling may not catch the full diversity of bacteria found in deeper layers [21]. A wound site was selected for wound headspace (defined as the airspace overlying the wound site) metabolite sampling as detailed below (selection of the wound site was varied between participants to equally distribute the sites sampled). The biopsy wound sites were dressed with Kaltostat (ConvaTec, Middlesex, UK), gauze and Tegaderm + pad dressing (3M, Minnesota, USA). Participants were asked to ensure the dressings remained *in situ* for 48 hours only and then no further dressings were required and wounds were left exposed to the air for the rest of the study. No sutures were required following the biopsy procedures for any of the participants. On day 7, wound headspace metabolite sampling and microbial sampling of one of the previous wound sites (the same site for both) were undertaken as described below (selection of the wound site was varied between participants to equally distribute the sites sampled). Additionally, objective non-invasive measures were performed at each visit for all participants to monitor the progression of normal wound healing as described below. The process was repeated at a different wound site on days 14, 21 and 28.

#### Wound headspace metabolome

Sample collection and processing. The participant’s local environment was assessed for signs of significant exogenous metabolite contamination and other clinical staff and patients were excluded from the sampling locality to reduce exogenous sample contamination. Previous studies have identified the composition of skin metabolites are influenced by the application of skin hygiene products [22], therefore, participants were advised not to use such products 24 hours prior to sampling. Custom designed 3D printed polylactic acid funnels were used to create a temporary headspace over the sampling site. These funnels allowed a tight seal at one end into which a thermal desorption (TD) tube could be inserted and the other end was a 10 ml volume chamber which would cover the sampling zone. TD tubes containing Tenax/Unicarb sorbent (Markes’ International, Llantrisant, UK) were attached to the funnel insert at one end and the headspace chamber placed over the wound site and held in place for 5 min, following which a 100 ml air sample was drawn through the TD tube using a piston hand pump (LP-1200, Honeywell analytics, Poole, UK). TD tubes were then resealed and stored at room temperature to await analysis. Un-opened TD tubes were also kept with the sampled tubes to act as environmental blanks.

Gas chromatography-mass spectrometry (GC-MS) analysis. Tenax^®^ TA tubes were conditioned at 300°C for 60 min with a nitrogen flow of 60 mL/min using a Markes TC-20 tube conditioner. Metabolites were desorbed from sorbent tubes at 280°C for 10 min, cryo-focussed on a cold trap maintained at -30°C and desorbed from the cold trap onto the GC (PerkinElmer Clarus 680) column by flash heating to 300°C for 2 min with a flow path temperature of 225°C. The GC column (BP1, 50 m x 0.22 mm [1 μm], SGE Analytical) was held at an initial temperature of 50°C for 1 min then ramped to 245°C at 5°C/min. The GC run time was 40 min with a total TD cycle time of 56 min. The PerkinElmer Clarus 600S mass spectrometer was in electron ionisation mode set at 70 eV. The source temperature was set to 180°C, and spectra were acquired in dynamic range extension mode at 3 scans/s over a range of 25–300 *m/z*.

Quantification was by reference to the response factor for toluene obtained by analysis of TD tubes loaded with 100 ng toluene as part of the same sequence as the sampled tubes. This compound was used as an internal standard (IS) due to exhibiting similar chemical properties and vapour pressure to the analytes of interest and also being not naturally found in the samples. Loading of toluene onto the TD tubes was undertaken using standard atmosphere equipment. Anhydrous toluene (Sigma Aldrich 99.8%) was evaporated at a controlled rate into a flow of clean air using a syringe driver. Mass flow controllers were used to control the dilution air flow and the air flow over the TD tubes in order to achieve the required loading level.

Data processing and analysis. GC-MS data were acquired and analysed using Masslynx (Waters Corp, Manchester, UK). Chromatographic peaks and mass spectra were cross-referenced with National Institute of Standards and Technology (NIST) library 14 for putative identification purposes. Chromatographic peaks were mass quantified as toluene.

#### Wound tissue microbiome

Sample collection. On day 7, one 5 mm diameter wound biopsy was performed encompassing one of the previous wound sites following headspace sampling. The above procedure was repeated at days 14, 21 and 28 encompassing a different wound site. The samples were stored at -80°C without any medium immediately following collection. The biopsy wound sites were dressed with Kaltostat (ConvaTec, Middlesex, UK), gauze and Tegaderm + pad dressing (3M, Minnesota, USA). Participants were asked to ensure the dressings remained *in situ* for 48 hours only and then no further dressings were required and wounds were left exposed to the air for the rest of the study. Additionally, all wounds were monitored at each visit. No sutures were required following the biopsy procedures for any of the participants.

Bacterial DNA isolation. Skin tissue from day 0 and wound biopsies were disrupted and homogenised using the Qiagen TissueRuptor (QIAGEN, Valencia, CA) according to the manufacturer’s recommendations. DNA was extracted using Qiagen DNEasy Blood and Tissue kit (QIAGEN, Valencia, CA) according to the manufacturer’s recommendations. Eluted DNA quantity and purity was assessed using Qubit (Invitrogen, Paisley, Renfrewshire, UK).

Sample sequencing and processing. Sequencing libraries were prepared as described in Illumina’s 16S Metagenomics Sequencing Library Preparation protocol Part # 15044223 Rev. B (Illumina Inc., San Diego, California, USA), targeting the V1-V3 region, with the following modifications. The samples were amplified to generate a 450bp product covering the V1-V3 regions of the 16S ribosomal subunit using nested primers (forward primer: 5’-AGATCGGAAGAGCACACGTCTGAACTCCAGTCAC-3, reverse primer: 5′-AGATCGGAAGAGCGTCGTGTAGGGAAAGAGTGTA-3). Following initial amplification, the libraries were validated using the Agilent BioAnalyzer2100 Instrument using the DNA1000 assay (Agilent Technologies, Wilmington, DE), and cleaned as outlined in the protocol. Illumina dual indexes and sequencing adapters were added using the following primers (Forward primer: 5’- AATGATACGGCGACCACCGAGATCTACAC [55555555]TCGTCGGCAGCGTC-3’, Reverse Primer: 5’- CAAGCAGAAGACGGCATACGAGAT[77777777]GTCTCGTGGGCTCGG-3’, where bracketed sequences are equivalent to the Illumina Index adapters N501-N508 and N701-N712). Following PCR, DNA was cleaned as outlined in the protocol. The libraries were further validated using the Agilent BioAnalyzer2100 Instrument using the DNA1000 assay. The samples were also quantified using the Broad Range Qubit Assay (Invitrogen, Paisley, Renfrewshire, UK).

Sequencing was performed on the Illumina MiSeq. Sequencing of the V1-V3 region was performed using 250 bp paired-end chemistry per sample. Data were converted to Bcl2FastQ. FastQ is a format that provides per-base quality scores additionally to the called bases. These scores reflect the confidence for accuracy of a given base-call. A total of 12,401,250 raw sequencing reads were produced on the MiSeq with a range of 170,246–1,069,137 sequences per sample. Raw fastq files were trimmed to remove Nextera i5 and i7 adapters and filtered to only include hiqh quality (>Q30: error probability ≤ 0.001) reads using Trimmomatic (version 0.36). OTUs were picked using the QIIME pipeline, using a 97% sequence similarity threshold, which is normally considered an appropriate proxy for species level identification. A total of 60,717 OTUs were identified from 5,473,747 high‐quality sequences from 25 samples. The GreenGenes (version 13_8) reference database was used for taxonomic assignment.

#### Objective non-invasive quantitative wound measurement devices

Objective non-invasive modalities as outlined below were used at each time point to monitor the progression of wound healing.

Spectrophotometric intracutaneous analysis. SIAscopy (MedX Health Corp, Mississauga, Canada) is a non-invasive technique which uses light reflected from the skin and performs quantitative measurements of haemoglobin, melanin, and collagen concentration and distribution [23]. SIAscopy operates by probing the skin spectrally over an area of 12 x 12 mm and at a depth of 2 mm.

Full-field laser perfusion imaging. Full-field Laser Perfusion Imaging (FLPI; Moor Instruments Ltd., Axminster, United Kingdom) is a laser doppler imaging technique, which measures blood flow in the skin’s microcirculation. This device uses low power light from a monochromatic stable laser and this is applied to skin/wound which becomes scattered by moving red blood cells which broadens the frequency [24]. This is then photo detected and is processed to provide an arbitrary blood flow measurement known as “flux”. This is proportional to the speed and concentration of red blood cells in the tissue [24].

Optical coherence tomography. Optical coherence tomography (OCT; VivoSight, Michelson Diagnos-tics Ltd, Kent, UK) is a non-invasive real-time tomographic imaging technique using low-intensity infrared light focused within living tissue. OCT provides depth-resolved images of tissues up to 2 mm deep with lateral resolution of 1 μm in some devices via a handheld instrument placed in contact with the skin/wound [25,26]. It can accurately delineate wound re-epithelialization, reformation of the dermo-epidermal junction, thickening of newly formed epidermis and dermal remodelling [27]. The VivoSight OCT also enables the visualisation of blood vessel formation (micro-circulation in the skin) when the dynamic mode is selected in the software.

Dermalab system. The DermaLab system by Cortex Technologies (Courage and Khazaka Electronic GmbH, Koln, Germany) is a multi-probe non-invasive device allowing the quantitative measurement of trans-epidermal water loss (TEWL), skin/wound hydration, melanin and erythema.

#### Statistical analysis

For wound tissue microbiome and objective non-invasive measures, principal component analysis (PCA) was applied to the data for visualisation. PCA is a commonly used technique for the dimensionality reduction of multivariate data whilst preserving most of the variance [28]. Principal component–discriminant function analysis (PC–DFA) was then used for further multivariate exploration of wound tissue microbiome data as PCA revealed poor separation. PC-DFA allows for the reduction of noise in the data without reducing relevant information from the original dataset [28]. Where applicable, for univariate analysis of the wound tissue microbiome and objective non-invasive measures, the non-parametric Kruskal–Wallis test and Wilcoxon signed ranks test was performed. Dunn-Bonferroni post-hoc test was then subsequently used to investigate statistically significant entities to account for multiple testing. The similarity between the wound tissue microbiome and objective non-invasive measures data sets was measured by using Procruestean test [29]. Procrustes analysis is an effective approach for assessing the similarities and differences between different ordination spaces from cluster analyses and has been used previously for the assessment of different analytical techniques [30]. First, one particular data set was chosen as target data and another data set was chosen as matching data. The matching data were optimally superimposed onto the target by using Procrustes rotation. The difference between the target and rotated matching data was measured by using normalized Procrustes distance. Normalized Procrustes distance varied from 0 to 1, 0 means a perfect match while 1 means nothing in common. To assess the statistical significance of such difference, a NULL distribution of normalized Procrustes distances were calculated using a series permutation tests in which the order of the target data set was randomly permuted and then calculate the normalized Procrustes distance between the permuted target and matching data. A total number of 10,000 permutations were performed and the Procrustes distances were calculated, recorded and formed the NULL distribution. An empirical *p*-value was derived by counting the number of cases when the Procrustes distances in the NULL distribution were lower than that between the target and matching data without permutation and divide it by the total number of permutations (i.e. 1000). If the Procruestean test showed non-significance, then Spearman’s correlation coefficient was calculated to assess the relationship between data sets. A *p*-value of <0.05 was considered statistically significant. Statistical analyses were performed in R, SPSS for Windows version 22.0 (SPSS, IBM, Armonk, NY, USA) and GraphPad Prism 7 (GraphPad Software, La Jolla, CA, USA).

### Part 2

#### Study participants

Healthy participants were enrolled into the study at the Manchester University NHS Foundation Trust, England, UK October and November 2017. The University of Manchester research ethics committee and Manchester University NHS Foundation Trust research and development department approval were granted for the study (Ethics number: 16045). All participants deemed suitable for enrolment (inclusion and exclusion criteria outlined in S1 Table), provided written informed consent. Six male participants were recruited, with an age range between 21–26 years.

#### Study design

On day 0, participants had two 4 mm diameter full thickness skin biopsies, performed under local anaesthetic (1% Lidocaine), in each of their upper inner arms leading to the creation of four iatrogenic wounds. Full thickness was defined as removing the entire epidermis and dermis to expose hypodermic fat. The punch biopsies were performed 5 cm from the axillary hairline and parallel to the medial epicondyle and were 3 cm in distance from each other. Pressure was applied to each biopsy site until haemostasis had been achieved. The biopsies were processed for metabolite profiling as detailed below. A wound site at random was selected for headspace metabolite sampling as detailed below. The biopsy wound sites were dressed with Kaltostat (ConvaTec, Middlesex, UK), gauze and Tegaderm + pad dressing (3M, Minnesota, USA). Participants were asked to ensure the dressings remained *in situ* for 48 hours only and then no further dressings were required and wounds were left exposed to the air for the rest of the study. No sutures were required following the biopsy procedures for any of the participants. On day 7, headspace and tissue metabolite sampling of one of the previous wound sites was undertaken as described below (selection of the wound site was varied between participants to equally distribute the sites sampled). Additionally, objective non-invasive measures were performed at each visit for all participants to monitor the progression of normal wound healing. The process was repeated at a different wound site on days 14, 21 and 28.

#### Wound headspace metabolome

Sample collection and processing. PDMS skin‐sampling patches measuring 20 mm × 15 mm × 0.45 mm (Goodfellow Cambridge Ltd., Huntingdon, UK), were washed in a solution of 5% Decon 90, followed by clean water then methanol, and conditioned in a stream of dry nitrogen for 1 hour at 350°C using a Markes TC20 conditioner (Markes International, Llantrisant, UK) before being stored in inert coated ¼” stainless steel tubes sealed with brass caps (Markes International). Prepared patches were stored at room temperature for a maximum period of 24 h before being transported to the participant. Participants were advised not to use skin hygiene products 24 hours prior to sampling and the local environment was assessed for signs of significant exogenous metabolite contamination; other clinical staff and patients were excluded from the sampling locality to reduce exogenous sample contamination. A PDMS patch was placed into a non-adherent dressing enveloped within aluminium foil located 1–2 m from the participant using sterile forceps. This was exposed for 30 min, providing a baseline measurement of the environmental metabolites present during sampling. Concurrently, two PDMS patches were placed within a non-adherent dressing 3 cm apart so that one covered the wound site and the other covered normal healthy skin. The non-adherent dressing was covered with aluminium foil and kept in situ using a lightly applied tourniquet for 30 min. After this time patches were removed from the sampling sites and resealed in stainless steel tubes. The samples were immediately transferred for analysis within 24 h of sample collection. During shipping and sampling, an additional patch remained sealed in a stainless tube to act as an analytical blank sample.

GC-MS analysis. Metabolite analysis was conducted on a thermal desorption-gas chromatography time-of-flight mass spectrometer (GC-TOF-MS) platform (Unity II TD with UltrA autosampler, Markes International, and Micromass GCT Premier, Waters Corp, Manchester, UK). Prior to desorption, 100 μl of an internal standard (IS; 1 ppmV 4-bromofluorobenzene in N_2_; Thames Restek, Bucks, UK) was loaded onto each tube. This compound was used as an IS as it exhibits similar chemical properties and vapour pressure to the analytes of interest whilst it is not naturally present in the samples, with previous use in clinical studies [31]. Metabolites were desorbed from TD tubes at 280°C for 5 min, cryo-focussed on a cold trap maintained at 0°C and desorbed from the cold trap onto the GC (Agilent 6890N) column by flash heating to 330°C for 3 min with a flow path temperature of 200°C. The GC column (DB-5MS column, 30 m, 0.25 mm internal diameter, 0.25 μm film thickness, Agilent) was held at an initial temperature of 40°C for 2 minutes, ramped to 250°C at 5°C min^-1^ and held for 16 min. The GC runtime was 60 min with a total TD cycle time of 65 min. The TOF-MS was in electron ionisation mode set at 70 eV with a source temperature of 200°C and a trap current of 100 μA, and spectra were acquired over a range of 40–500 *m/z*.

Data processing and analysis. The GC-MS raw files were firstly converted to mzXML and subsequently imported to R [32]. A R package “erah” was employed to de-convolve the GC-MS files [33,34]. A total number of 129 unique peaks were detected in the wound headspace metabolome data. Chromatographic peaks and mass spectra were cross-referenced with NIST library 14 for putative identification purposes, and followed the metabolomics standards initiative (MSI) guidelines for metabolite identification [35]. The peak intensities were log_10_-scaled before further statistical analysis.

#### Wound tissue metabolome

Sample collection and processing. On day 7, one 5 mm diameter wound biopsy was performed encompassing one of the previous wound sites. The above procedure was repeated at days 14, 21 and 28 encompassing a different randomly selected wound site. The samples were stored at -80°C without any medium immediately following collection. The biopsy wound sites were dressed with Kaltostat (ConvaTec, Middlesex, UK), gauze and Tegaderm + pad dressing (3M, Minnesota, USA). Participants were asked to ensure the dressings remained *in situ* for 48 h only and then no further dressings were required and wounds were left exposed to the air for the rest of the study. No sutures were required following the biopsy procedures for any of the participants.

Metabolite extraction involved suspending wound samples in 1.2 ml of methanol (80%) solution at– 20°C and homogenising with a steel bead for 20 min at 25 Hz. Samples were then vortex mixed for 15 s, followed by centrifugation at 8000 *g* for 10 min. Aliquots (900 μL) of wound supernatant extracts were normalised according to sample biomass. Following published protocols [36], 100 μl aliquots from each sample were combined to be used as pooled quality control (QC) samples. Subsequently, 100 μl of IS solution (0.2 mg/ml succinic-*d*_4_ acid, and 0.2 mg/ml glycine-*d*_5_) was added to all the samples (including QCs) and vortex mixed for 15 s. Samples were lyophilised by speed vacuum concentration at room temperature for 16 h (HETO VR MAXI vacuum centrifuge attached to a Thermo Svart RVT 4104 refrigerated vapour trap; Thermo Life Sciences, Basingstoke, U.K.). A two-step derivatization protocol of methoxyamination followed by trimethylsilylation was employed [37]. The extracts were re-dissolved in 50 μl of 20 mg.mL^-1^ O-methoxylamine hydrochloride in pyridine, vortex mixed, and incubated at 60°C for 30 min in a dri-block heater. Subsequently, 50 μl of N-methyl-N-(trimethylsilyl) trifluoroacetamide (MSTFA) was added and the extracts incubated at 60°C for a further 30 min. On completion, 20 μl of retention index solution was added (0.3 mg/mL n-docosane, n-nonadecane, n-decane, n-dodecane, and n-pentadecane in pyridine) for chromatographic alignment prior to centrifugation at 13000 *g* for 15 min. The resulting supernatant (120 μl) was transferred to GC-MS vials for analysis.

GC-MS analysis. GC-MS analysis was conducted on a 7890B GC coupled to a 5975 series MSD quadrupole mass spectrometer and equipped with a 7693 autosampler (Agilent, Technologies, UK). The sample (1 μL) was injected onto a VF5-MS column (30 m x 0.25 mm x 0.25 μm; Agilent Technologies) with an inlet temperature of 280°C and a split ratio of 20:1. Helium was used as the carrier gas with a flow rate of 1 mL/min. The chromatography was programmed to begin at 70°C with a hold time of 4 min, followed by an increase to 300°C at a rate of 14°C/min and a final hold time of 4 min before returning to 70°C. The total run time for the analysis was 24.43 min. The MS was equipped with an electron impact ion source using 70 eV ionisation and a fixed emission of 35 μA. The mass spectrum was collected for the range 50–550 *m/z* with a scan speed of 3,125 (N = 1). Samples were analysed in a randomised order with the injection of a pooled biological quality control sample after every 6^th^ sample injection.

Data processing and analysis. The GC-MS raw files were firstly converted to mzXML and subsequently imported to R [32]. A R package “erah” was employed to de-convolve the GC-MS files [33,34]. A total number of 346 unique peaks were detected in the wound tissue metabolome data. Chromatographic peaks and mass spectra were cross-referenced with the Golm library for putative identification purposes, and followed the metabolomics standards initiative (MSI) guidelines for metabolite identification [35]. The peak intensities were log_10_-scaled before further statistical analysis.

#### Objective non-invasive quantitative wound measurement devices

Objective non-invasive imaging modalities as outlined above were used at each time point to monitor the progression of wound healing.

#### Statistical analysis

For wound headspace metabolome, wound tissue metabolome and non-invasive measures, PCA was applied to the data for visualization. For wound headspace metabolome, ANOVA-simultaneous component analysis (ASCA) was then employed to reveal the effect of each factor under study separately, i.e. time and sampling site [38]. Although, PCA is a popular tool for exploratory analysis, it is not as effective when the study comprises multiple influential factors. Therefore, as the wound headspace metabolome contained multiple factors (site and time points), ASCA was utilised to analyse these data. For univariate analysis of the wound headspace metabolome, Friedman test was applied to each of the peaks to detect features which changed significantly between different time points and sampling site individually. For each *p*-value, a false discovery rate (FDR) was also calculated using Benjamini and Hochberg procedure. For univariate analysis of the wound tissue metabolome and objective non-invasive measures, the non-parametric Kruskal–Wallis test was performed. Dunn-Bonferroni post-hoc test was then subsequently used to investigate statistically significant entities. The similarity between the wound tissue microbiome or the wound headspace metabolome and objective non-invasive measures data sets was measured by using Procruestean test. A *p*-value of <0.05 was considered statistically significant. Statistical analyses were performed in R, SPSS for Windows version 22.0 (SPSS, IBM, Armonk, NY, USA) and GraphPad Prism 7 (GraphPad Software, La Jolla, CA, USA).

## Results

### Wound tissue metabolome significantly varied between time points

**Fig 1:**
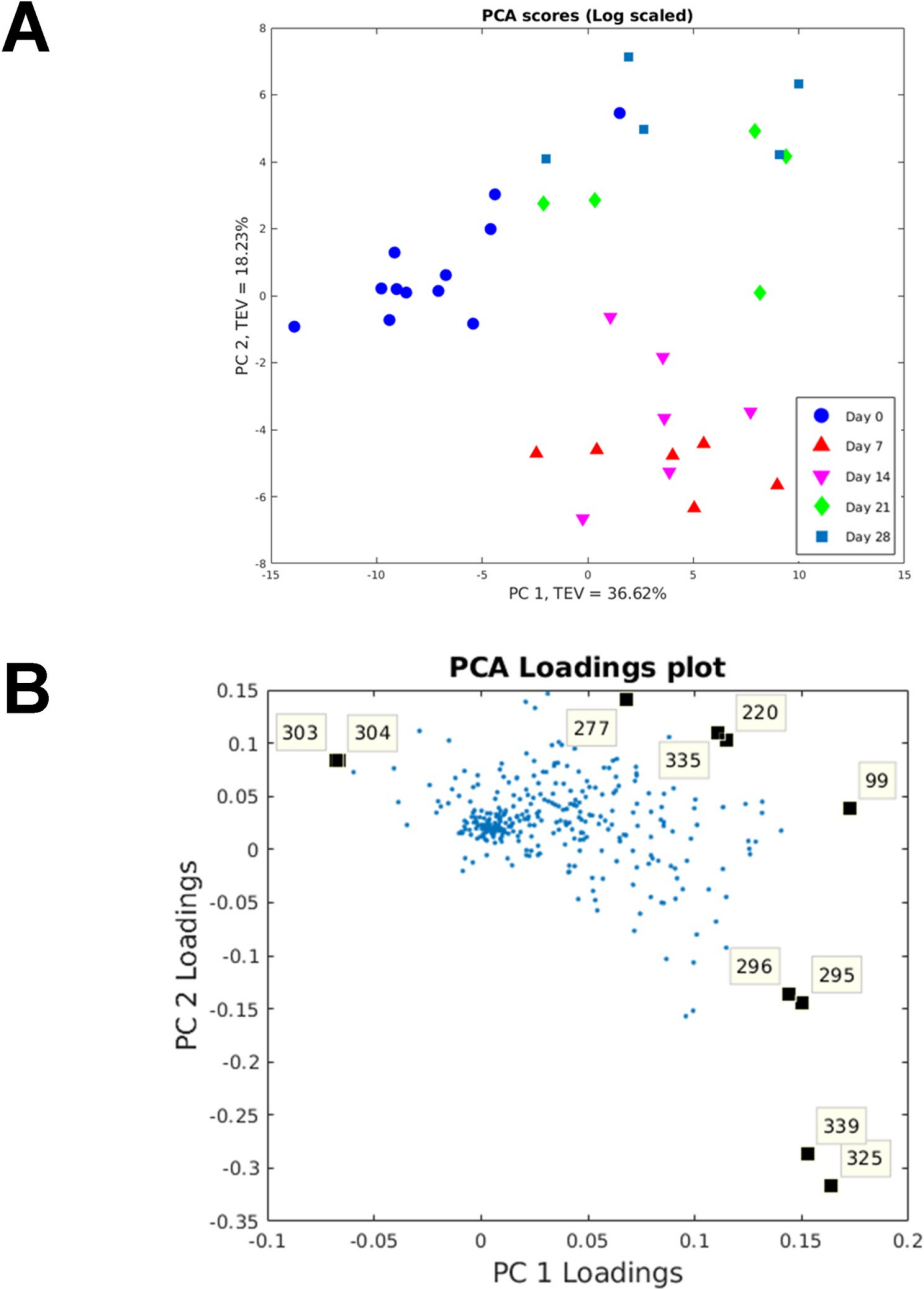
Principal component analysis (PCA) and PCA loadings on wound tissue metabolome. (A) PCA scores plot of principal component (PC) 1 vs. PC2. The total explained variance of PC1 is 36.62% and for PC2 is 18.23%. (B) PCA-loadings plot: 99—unknown; 220—l-glutamine; 277–1,3-dihydroxyacetone dimer; 295 and 296—linolenic acid; 303 and 304—unknown; 325 and 339—glycerol; 335—adenosine.

**Fig 2:**
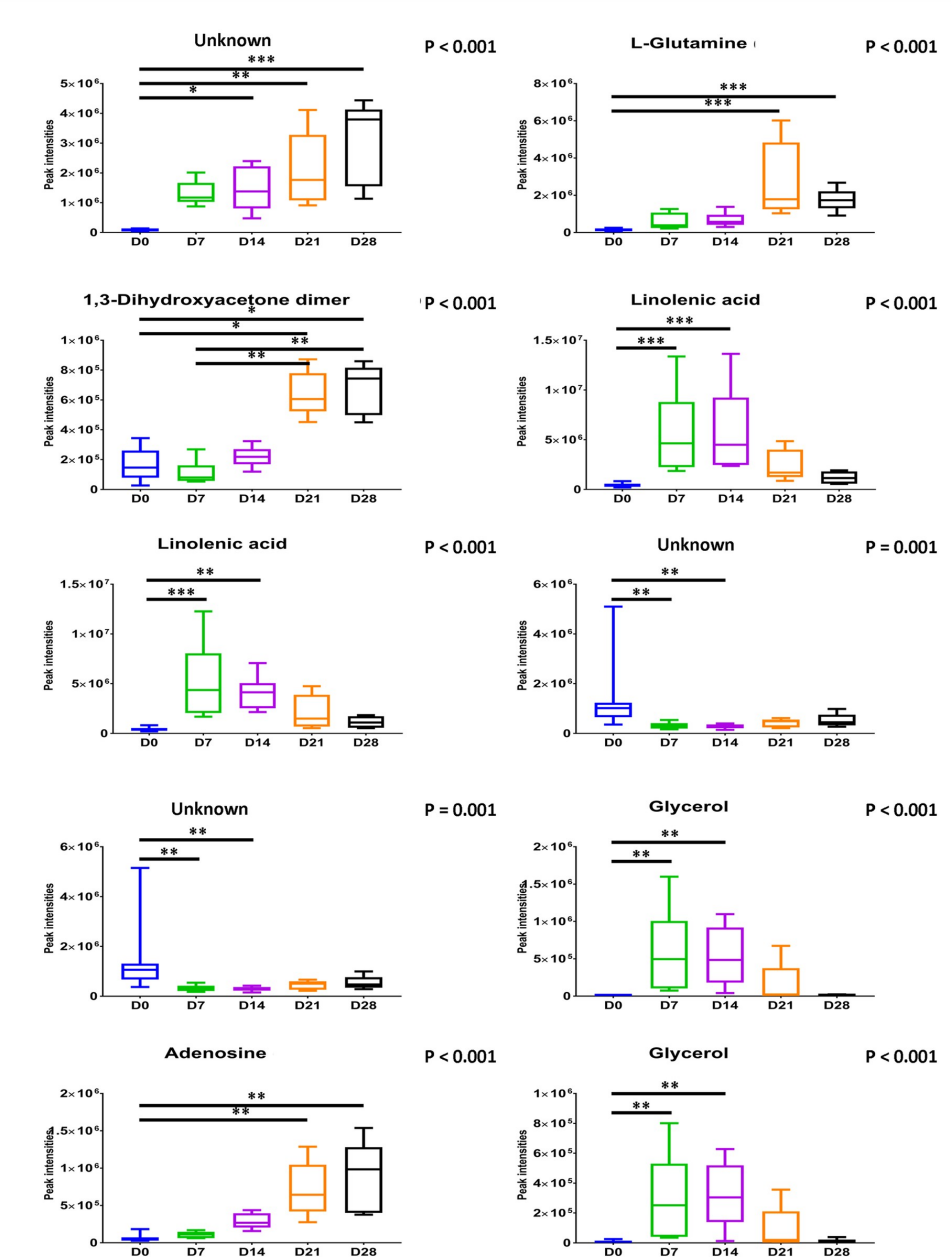
Boxplots showing significantly varied metabolites as identified from multivariate analysis across time points. Kruskal–Wallis test with accompanying Dunn-Bonferroni post hoc analyses were performed (*n* = 6). * *P* < 0.05, ** *P* < 0.01, *** *P* < 0.001.

Many metabolite features (*n* = 346) were detected using the wound tissue sampling method. The compounds were tentatively identified using the mass spectral library and have not been confirmed using analytical standards [35]; we therefore consider these to be identified to level 2 of Metabolomics Standards Initiative (MSI) as they are from gas chromatography-mass spectrometry (GC-MS) database matches to the Golm and National Institute of Standards and Technology (NIST) 14 library. Principal component analysis (PCA) was used to visualise the changes in metabolites that can be used to differentiate wound healing time points (Fig 1A). There was clear differentiation between day 0 and the other time points on the principal component (PC) 1 axis with a total explained variance (TEV) of 36.62%. Day 7 and 14 wound samples were closely clustered as were day 21 and 28. There was clear differentiation between these two clusters on the PC2 axis with a TEV of 18.23%. PCA loadings plot identified the specific metabolites responsible for the separation (Fig 1B). From the loadings plot: l-glutamine, 1,3-dihydroxyacetone dimer, linolenic acid, linolenic acid, glycerol, glycerol, adenosine and three unknowns were found to be contributing metabolites to separation of the time points. All the metabolites suggested by the PCA above were found to significantly vary across the time points (*P*≤0.001, Kruskal–Wallis test with accompanying Dunn-Bonferroni post hoc analyses; Fig 2).

### Wound headspace metabolome significantly varied between sampling sites and time points

**Fig 3:**
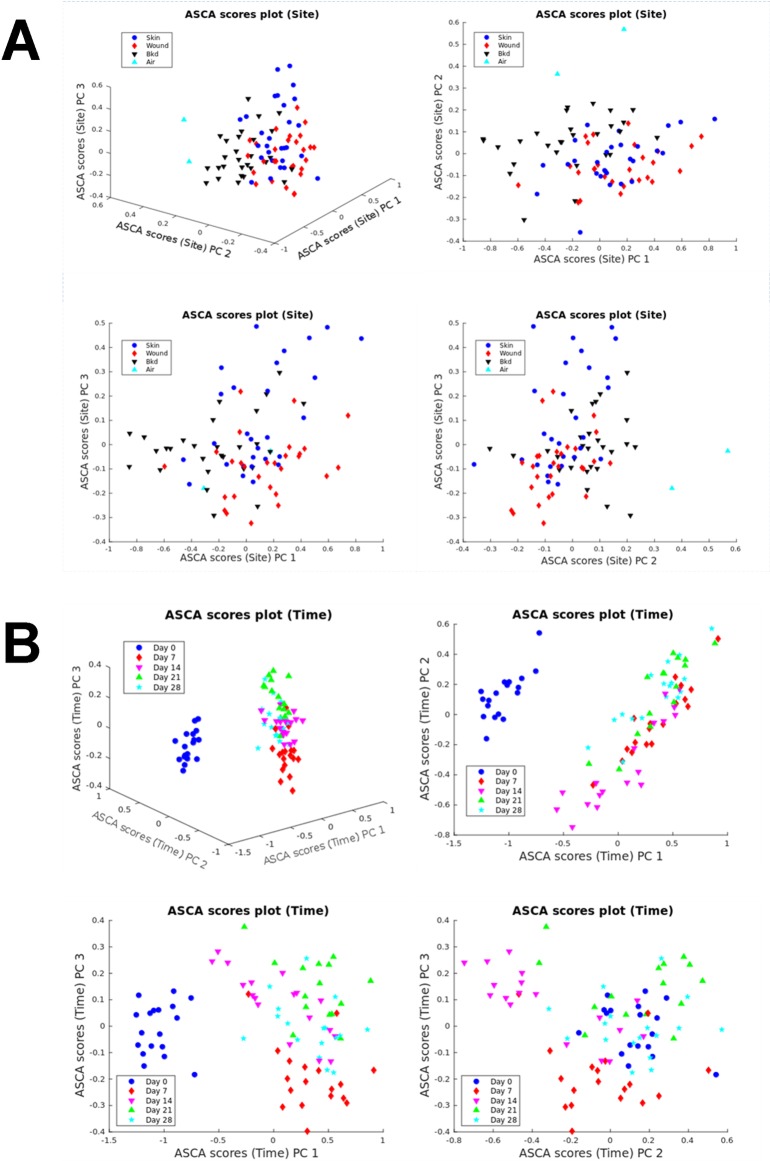
Analysis of variance–simultaneous component analysis (ASCA) on wound headspace metabolome. (A) ASCA scores plot for site. (B) ASCA scores plot for time.

**Fig 4:**
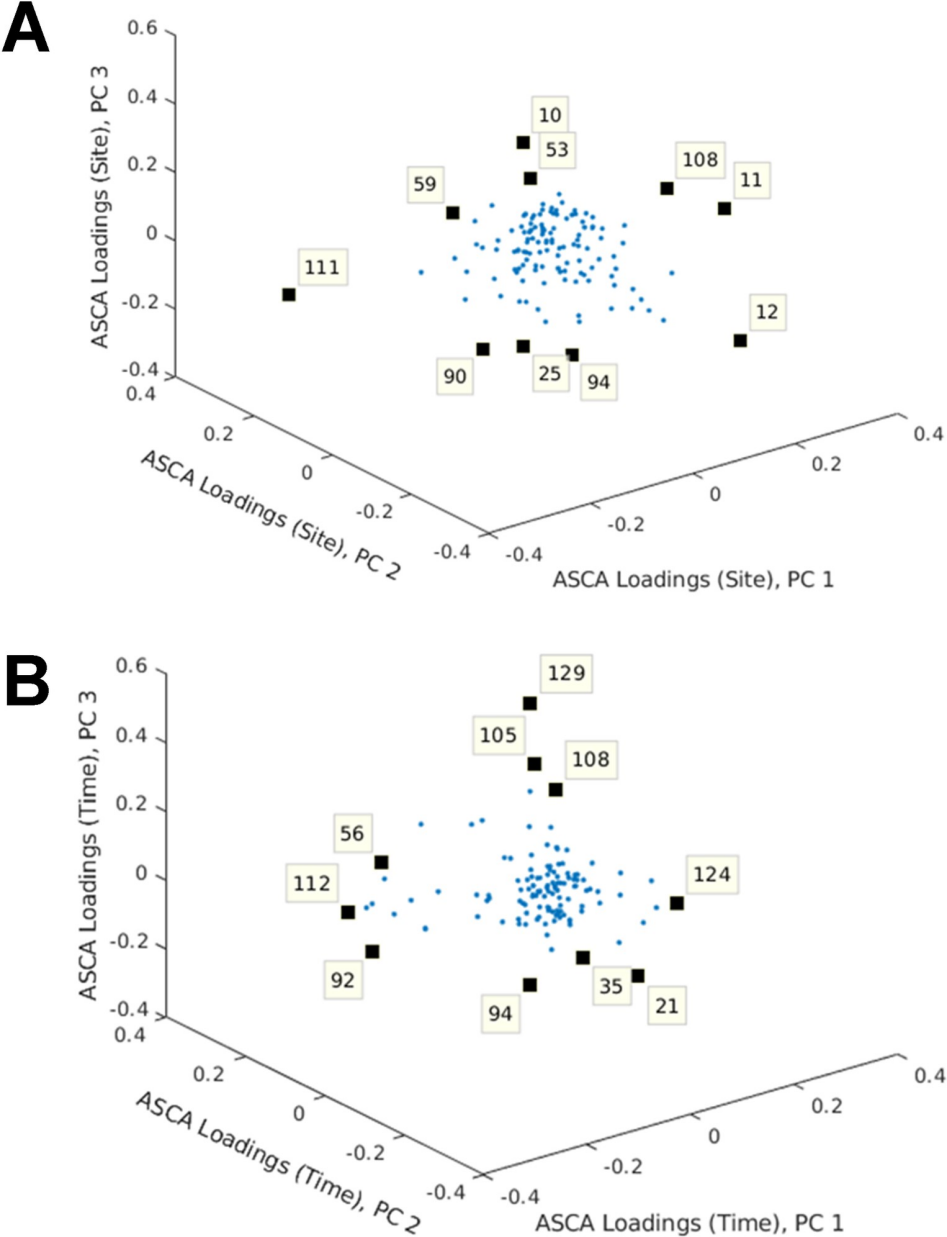
Analysis of variance–simultaneous component analysis (ASCA) loadings plot for wound headspace metabolome. (A) ASCA loadings plot for site: 10, 11, 12, 25, 53, 59, 90, 94, 108 –unknown; 111–1,3-bis(1,1-dimethylethyl)benzene. (B) ASCA loadings plot for time: 21, 35, 56, 92, 94—unknown; 105–1,5-dimethyl-2-oxabicyclo[3.2.1] nonan-7-one; 108—unknown; 112–1,3-bis(1,1-dimethylethyl) benzene; 124 –unknown; 129—isobutyl-2,2,4-trimethyl-3-hydroxypentanoate.

As detection of the wound tissue metabolome requires invasive punch-biopsy sampling of wound tissue, a non-invasive polydimethylsiloxane (PDMS) patch method was implemented. This method required minimal sample preparation and therefore had superior clinical usability. Less metabolite features (*n* = 129) were detected using PDMS in comparison to the wound tissue metabolome method. PCA was used to visualise the changes in metabolites that could be used to differentiate wound healing time points (S2 Fig). There was clear separation between day 0 and the other time points on the PC1 axis with a TEV of 41.62%. However, there was no clear separation between the other time points and no clear differentiation between the sampling sites (i.e. between background, skin and wound). Although, PCA is a popular tool for exploratory analysis, it is not as effective when the study comprises multiple influential factors. Therefore, as the wound headspace metabolome contained multiple factors (site and time points), analysis of variance–simultaneous component analysis (ASCA) was utilised to analyse these data. There was a statistically significant difference in the wound headspace metabolome between sites but with considerable overlap between them (*P* = 0.0024; Fig 3A). There was also a statistically significant difference in the wound headspace metabolome between time points with the most obvious differences between day 0 and other time points (*P*<0.0001; Fig 3B). Day 7 also appeared to be rather different to the other time points with appreciable overlap between the remaining days (days 14–28). ASCA loadings plot identified the specific metabolites responsible for the separation between sites (Fig 4A) and time points (Fig 4B). According to the loadings plot (Fig 4A): 1,3-bis(1,1-dimethylethyl)benzene and nine unknowns were found to be contributing metabolites to separation of the sites. The greatest separation between skin and wounds was seen along PC3 (Fig 3A) with the unidentified metabolites contributing to this separation. From the loadings plot (Fig 4B): 1,5-dimethyl-2-oxabicyclo[3.2.1]nonan-7-one, 1,3-bis(1,1-dimethylethyl)benzene, isobutyl-2,2,4-trimethyl-3-hydroxypentanoate and seven unknowns were found to be contributing metabolites to separation of the time points.

Of the 10 metabolites detected from ASCA above, 1,3-bis(1,1-dimethylethyl)benzene significantly varied between sampling sites (*P*<0.05, Friedman test with a false discovery rate (FDR) calculated using Benjamini and Hochberg procedure). Moreover, all 3 identified metabolites were also found to significantly vary across the time points (*P*<0.05, Friedman test with a FDR calculated using Benjamini and Hochberg procedure).

### PDMS metabolite detection was superior to Tenax/Unicarb sorbent method

Only three metabolites (ethanol, acetone and propan-2-ol) were identified using the Tenax/Unicarb sorbent method. Therefore, no further statistical analyses was undertaken.

### Microbiome analysis identified the reciprocal relationship between *Staphylococcus* and *Propionibacterium*

**Fig 5:**
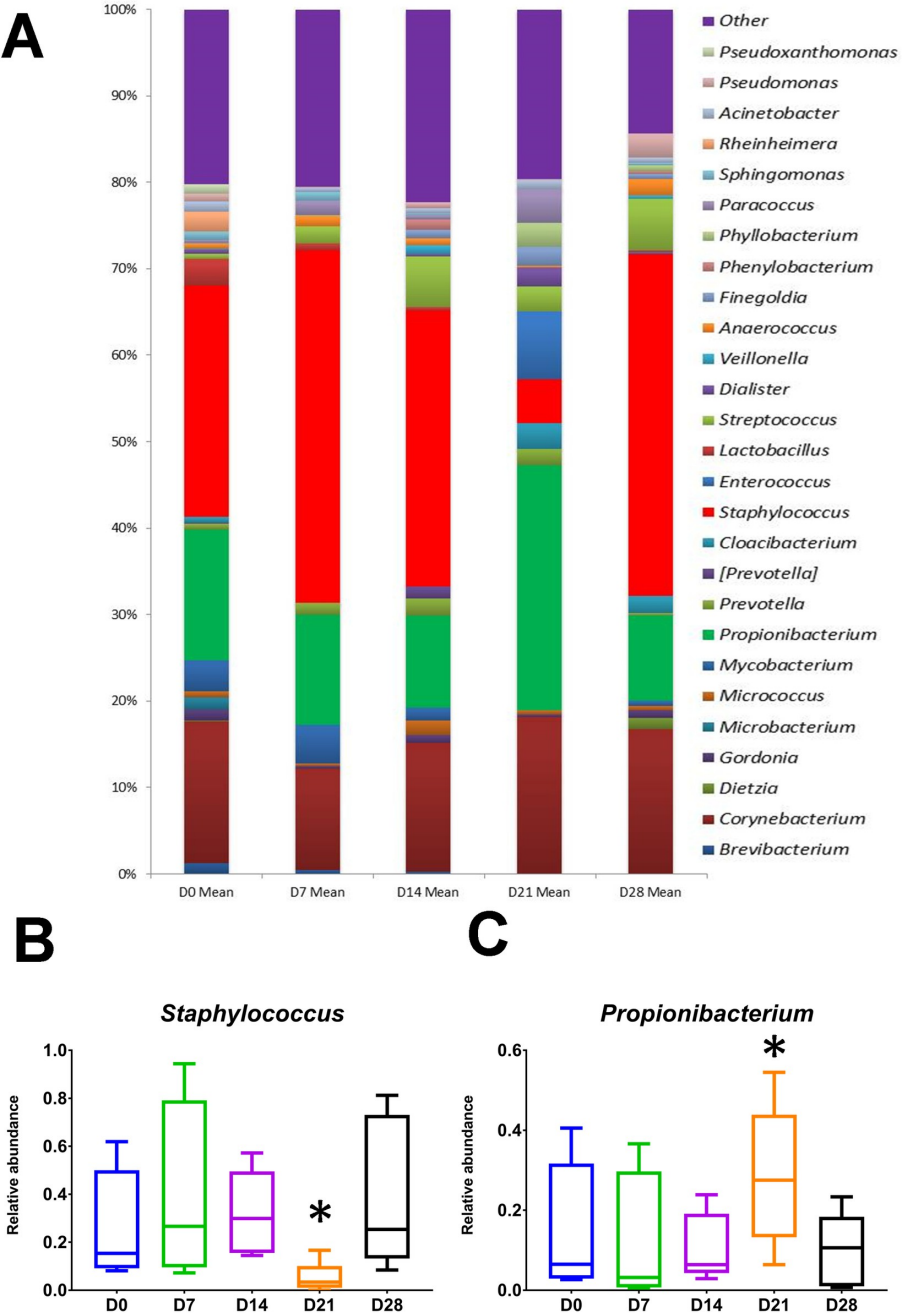
Wound microbiome. (A) Mean relative abundances of bacterial taxa between time points. Relative abundance is shown on the Y-axis. Taxa are filtered to those with a mean abundance greater than 1%. (B and C) Boxplots showing the relative abundances of *Staphylococcus* and *Propionibacterium* across time points. * denotes P < 0.05, as determined by Wilcoxon’s signed ranks test.

Fig 5A outlines the mean relative abundances of bacterial genera between time points. Of note was the reciprocal relationship between Staphylococcus and Propionibacterium at day 21 (*P*<0.05, Wilcoxon signed ranks test; Fig 5B and 5C). The relative abundances of *Brevibacterium*, *Microbacterium*, *Mycobacterium* and *Paracoccus* species significantly varied across the time points (S3 Fig).

Principal component analysis (PCA) was utilised to visualise the dynamic changes in the microbiome across the wound healing time points and attempt to identify the most discriminant bacterial genera that can be used to differentiate these time points (S4A Fig). There was no clear separation between the time points based on differences in the bacterial genera. Therefore, to maximise the variance between groups and minimise the variance within groups, principal component–discriminant function analysis (PC-DFA) was employed. Ten PCs, which accounted 97.4% variance of the dataset were included for discriminant function analysis (DFA) (S4B Fig). This however, showed no clear separation.

### Trans-epidermal water loss, haemoglobin, blood flow, melanin and attenuation compensation allowed differentiation of wound time points

#### Part 1

PCA was used to visualise the changes in non-invasive measures that can be used to differentiate wound healing time points (S5A Fig). There was clear differentiation between time points on the PC1 axis with a TEV of 27.84%. The greatest separation was seen between day 0 and 7 and there was a gradual return towards day 0 parameters from day 7 to day 28. There was also a clear differentiation between time points on the PC2 axis with a TEV of 18.75%. The greatest separation was seen between day 0 and day 21–28. PCA loadings plot identified the specific non-invasive measures responsible for the separation (S5B Fig). Trans-epidermal water loss (TEWL), haemoglobin and attenuation compensation were observed to contribute to the separation between day 0 and 7. Blood flow, melanin and attenuation compensation were observed to contribute to the separation of day 0 and day 21–28. All wounds had healed with 100% reepithelialisation by day 28. TEWL, erythema, haemoglobin, blood flow and attenuation compensation significantly varied across the time points (*P*<0.05, Kruskal–Wallis test with accompanying Dunn-Bonferroni post hoc analyses; S6 Fig).

#### Part 2

PCA was used to visualise the changes in non-invasive measures that can be used to differentiate wound healing time points (S7A Fig). There was clear differentiation between time points on the PC1 axis with a TEV of 39.84%. The greatest separation was seen between day 0 and 7 and there was a gradual return towards day 0 parameters from day 7 to day 28. PCA loadings plot identified the specific non-invasive measures responsible for the separation (S7B Fig). TEWL, blood flow, melanin, haemoglobin and attenuation compensation were observed to contribute to the separation between day 0 and 7. TEWL, erythema, haemoglobin, blood flow and attenuation compensation significantly varied across the time points (*P*<0.05, Kruskal–Wallis test with accompanying Dunn-Bonferroni post hoc analyses; S8 Fig). All wounds had healed with 100% reepithelialisation by day 28.

### Collagen correlated significantly with *Propionibacterium* and *Staphylococcus*

Procrustes analysis was performed using the non-invasive measures as a reference when assessing the similarities between the multivariate datasets of wound tissue microbiome and non-invasive measures (metadata). The Procrustes distance was 0.9161 (*P*>0.05) indicating poor similarity as a whole. Therefore, Spearman’s correlation coefficient was used to assess the relationship between objective non-invasive measures in univariate level and the wound tissue microbiome with significant correlations outlined in S2 Table. Of note, were the significant correlation between collagen and *Propionibacterium* and *Staphylococcus*; between blood flow and *Mycobacterium* and *Propionibacterium*; and between attenuation compensation and *Brevibacterium* and *Mycobacterium* (S9 Fig). Further species level analyses identified *Propionibacterium* genus consisted of *P*. *acnes* and *Staphylococcus* genus consisted of *S*. *aureus* and *S*. *epidermidis* along with other species (<1% relative abundance). *S*. *aureus* significantly correlated with collagen (r = -0.397; P<0.05), whereas, no such relationship was identified between *S*. *epidermidis* and collagen (r = -0.072; P>0.05).

### Non-invasive measures of wound healing clustered with wound headspace metabolites

**Fig 6:**
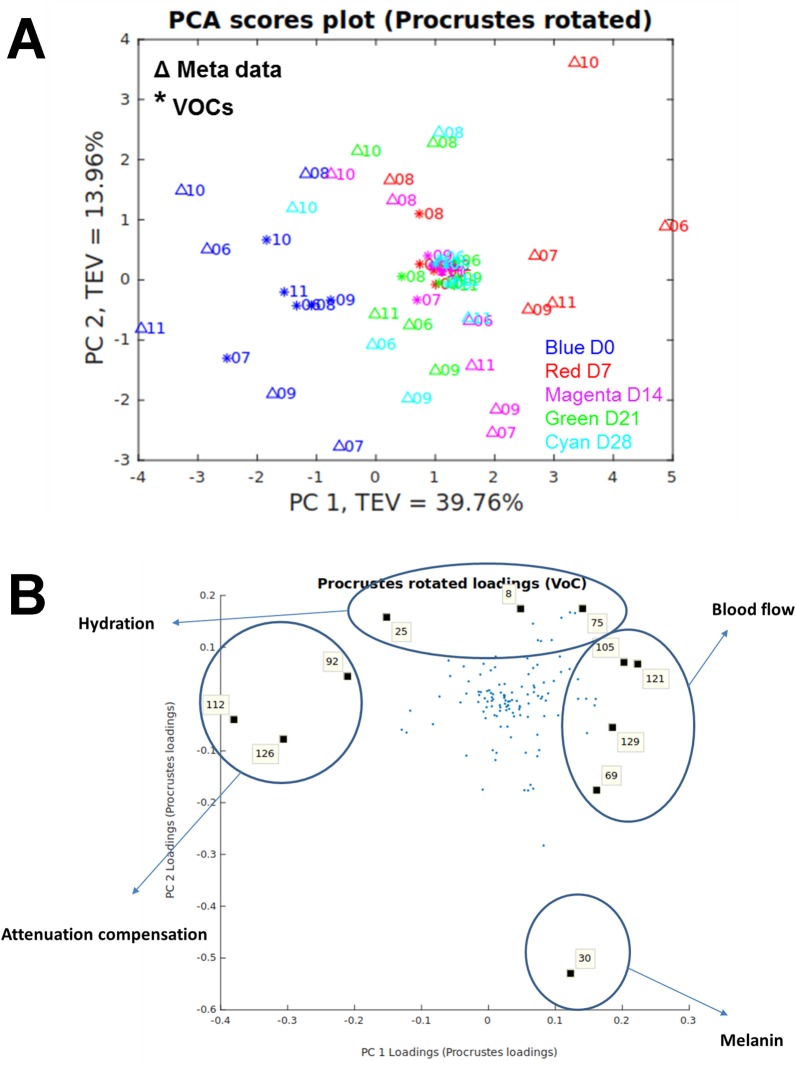
Procrustes analysis of non-invasive measures and wound headspace metabolome. (A) Procrustes rotated plot with a Procrustes distance of 0.7803; *P* = 0.0016. (B) Procrustes rotated loadings plot highlighting metabolites mostly correlated to the non-invasive measures. 8, 25, 30, 69, 75, 92—unknown; 105–1,5-dimethyl-2-oxabicyclo[3.2.1] nonan-7-one; 112–1,3-bis(1,1-dimethylethyl) benzene; 121, 126 –unknown; 129—isobutyl-2,2,4-trimethyl-3-hydroxypentanoate.

Procrustean test was performed to measure the similarity between these two data sets and the non-invasive measures data was used as a reference. The Procrustes distance was 0.7803 with a *P* value of 0.0016 (Fig 6A). The Procrustes rotated loadings plot identified the metabolites with significantly high loadings contributing most to the matched patterns (Fig 6B). Of the identified compounds: 1,5-dimethyl-2-oxabicyclo[3.2.1] nonan-7-one and isobutyl-2,2,4-trimethyl-3-hydroxypentanoate clustered with blood flow; and 1,3-bis(1,1-dimethylethyl)benzene clustered with attenuation compensation.

### Non-invasive measures of wound healing clustered with wound tissue metabolites

**Fig 7:**
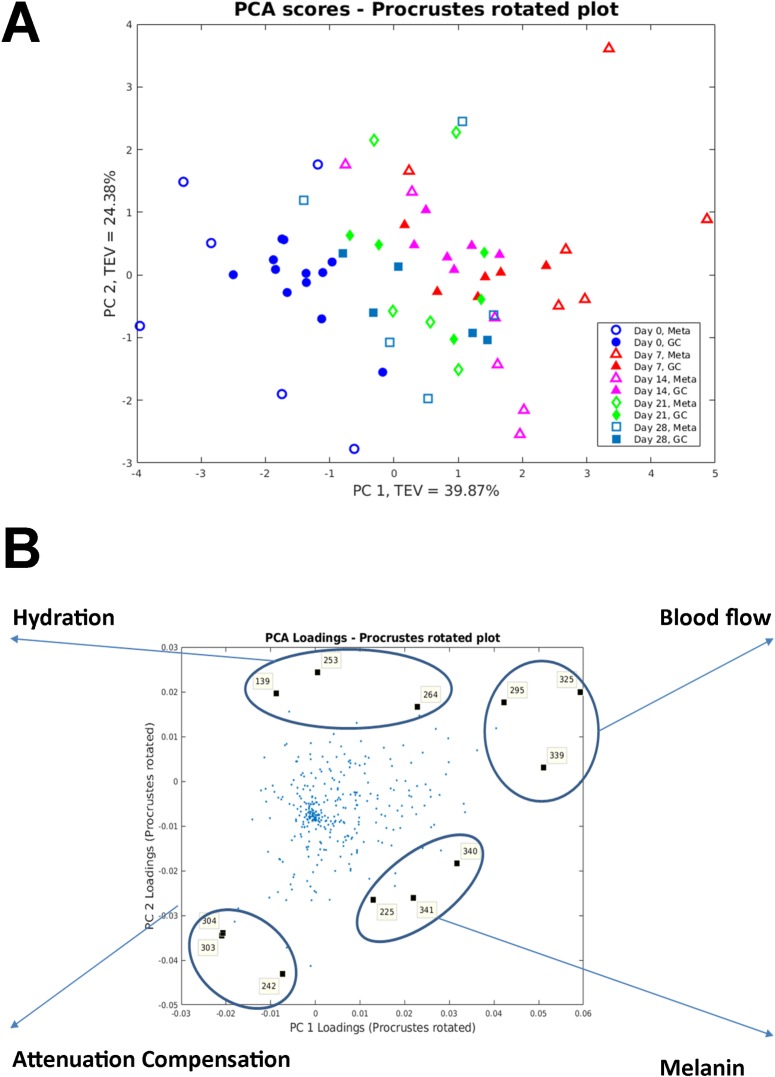
Procrustes analysis of non-invasive measures and wound tissue metabolome. (A) Procrustes rotated plot with a Procrustes distance of 0.6817; *P*<0.001. (B) Procrustes rotated loadings plot highlighting metabolites mostly correlated to the non-invasive measures. 139–1-methyladenosine; 225—linolenic acid; 242—D-(+)-galactose; 253—myo-inositol; 264 –xanthine; 295—linolenic acid; 303 and 304—unknown; 325 and 339—glycerol; 340—shikimic acid; 341—D-(+)-maltose.

Procrustean test was performed using the non-invasive measures as a reference. The Procrustes distance was 0.6817 with a *P* value of 0.001; Fig 7A). The Procrustes rotated loadings plot identified the metabolites with significantly high loadings contributing most to the matched patterns (Fig 7B). 1-methyladenosine, myo-Inositol and xanthine clustered with hydration; linolenic acid, glycerol and glycerol clustered with blood flow; D-(+)-galactose and two unknown metabolites clustered with attenuation compensation; and linolenic acid, shikimic acid and D-(+)-maltose clustered with melanin.

## Discussion

In this novel two part human exploratory study, we have demonstrated the complex temporal metabolome and microbiome profiles of the early phases of acute wound healing. To our knowledge this was the first multi-time point study investigating the dynamic metabolome and microbiome in acute wounds, with only three previous studies to date having explored the metabolic profile of chronic wounds at a single time point [11–13]. Here, we were able to statistically demonstrate the relationship between the metabolome and microbiome to acute wound healing processes. Using three methods of metabolite sampling, we have established the dynamic nature of the acute wound metabolome, as well as exploring the sensitivity of each technique. With the use of established next generation sequencing methods, we have outlined the changing nature of the acute wound microbiome. Finally, by utilising validated objective non-invasive measures of wound healing, we have successfully shown the relationships between the wound metabolome and microbiome to acute wound healing processes.

Using four non-invasive objective skin measurement devices, we quantified 12 parameters as objective measures of wound healing processes [39]. These are sub-characterised into blood flow (DermaLab erythema, Full-field Laser Perfusion Imaging, SIAoscopy Hb and optical coherence tomography (OCT) blood flow), skin barrier function (DermaLab TEWL and hydration), tissue morphology (SIAoscopy collagen, OCT attenuation compensation) and pigmentation (Dermalab melanin and SIAoscopy melanin). The attenuation compensation measured by OCT is the amount by which the optical signal reduces with the distance travelled into the wound and is related to collagen density and organisation. It is deemed to be a measure of extracellular matrix remodelling and fibrosis [40]. These parameters allowed for the quantitative measurements of acute healing phases, namely, inflammation, proliferation and remodelling. This allowed for the exploration of how these processes are related to the wound metabolome and microbiome.

The method of wound tissue sampling for metabolome analysis allowed for the detection of over three-hundred metabolite features across the five sampling time points. Multivariate PCA allowed visualisation of how the wound tissue metabolome changed across time with clear separation of the time points based on the metabolic profile with clustering of day 0, day 7–14 and day 21–28. From the PCA loadings plot, ten metabolites were identified as significant to this separation. Linolenic acid and glycerol were significant metabolites allowing differentiation between normal skin and early wounds with a significant increase in abundances at day 7 and 14. L-glutamine, 1,3-dihydroxyacetone dimer, adenosine and one unknown metabolite were significant metabolites allowing differentiation between early and later time points, suggesting their presence and abundance may represent a measure of varying wound maturity. Procrustes analysis identified relationships between wound tissue metabolites and wound healing processes. Linolenic acid was found to be related to blood flow. Linolenic acid is an essential fatty acid that is not produced within the body. However, along with its derivatives, studies have shown their beneficial effect on skin and healing [41]. Linolenic acid and its derivatives have been shown to modify the immune response, inhibiting the inflammatory response through inhibition of pro-inflammatory cytokines such as tumour necrosis-α and interleukin-12; and also promoting wound healing [42]. Linolenic acid has also been shown to improves cell migration [43], enhance cerebral vasodilatation [44] and induce angiogenesis [45]. Xanthine was related to wound hydration and this corroborates with Méchin *et al*. who proposed xanthine derivatives are able to correct the hydration of the epidermis [46]. Endogenous glycerol is a trihydroxy alcohol that has been shown to facilitate wound healing with specific roles in skin hydration, cutaneous elasticity and epidermal barrier repair [47]. D-(+)-galactose was associated with attenuation compensation, a marker of extracellular matrix formation and collagen deposition. This is supported by a previous rat based model that showed galactose based metabolites caused significant increases in the accumulation of granulation tissue [48]. We also reported, to our knowledge, the first instance of metabolites found to relate to wound healing processes, such as, glycerol with blood flow; 1-methyladenosine and myo-inositol with hydration; an unknown metabolite with attenuation compensation; and linolenic acid, shikimic acid and D-(+)-maltose with melanin. However, caution must be applied when interpreting these findings due to limited size of samples and the metabolites were only tentatively identified. Further confirmatory studies are required before firm conclusions can be drawn.

The Tenax/Unicarb sorbent method was used to sample the wound headspace metabolome in part one of the study, whereas in part two, this was switched to the PDMS sampling method. Wound headspace sampling of the metabolome has the advantageous benefit over wound tissue sampling as it is non-invasive and requires minimal sample preparation [49]. However, the sampling method used had a great impact on the data output. The Tenax/Unicarb sorbent method identified only three metabolites and therefore further sampling and analysis was abandoned. Therefore, the PDMS sampling method, which has been validated in skin and wound sampling [13,50], was employed for part two of the study. This technique identified a significant increase in the number of metabolite features detected compared to the Tenax/Unicarb sorbent method. This number however, was still only just over a third of metabolites identified through the wound tissue sampling method. Detection identification of 129 metabolite features and multivariate ASCA showed statistically significant differences of the metabolome sampling sites and also across time points. PDMS wound headspace sampling had the benefit over wound tissue sampling, as multiple site sampling (wound, skin and background) were able to be conducted, allowing greater comparative analyses. However, tentative identification was more difficult in matching wound headspace metabolite features to named compounds.

The headspace metabolome varied significantly between the sampling sites. However, due to limitations in metabolite identification, only 1,3-bis(1,1-dimethylethyl)benzene, a phenylpropane, was identified as a significant metabolite contributing to the separation. Human exposure to benzene mainly occurs through inhalation, oral and dermal routes and is predominantly deposited in fatty tissues [51]. 1,3-bis(1,1-dimethylethyl) benzene has been detected in multiple bio-fluids, such as faeces [52] and saliva [53], however, this was the first instance that it was detected in human skin and wounds. A possible explanation for the variation in the hydroxypentanoate and benzene derivatives between time points and sampling sites could be their relationship to changes in the wound microbiome. Both have previously been shown to be derived from or have an effect on the microbiota [54,55]. However, as the identification of metabolites is only tentative, these results must be taken with caution and further confirmatory studies are necessary. Procrustes analysis of wound headspace metabolites identified a relationship between 1,3-bis(1,1-dimethylethyl)benzene and attenuation compensation, a measure of tissue remodelling. A strong positive correlation has previously been identified between percentages of apoptotic cells and quantities of 1,3-bis(1,1-dimethylethyl)benzene [56]. Our identification of the association of this compound to the wound remodelling phase, therefore, could be due to its relationship to apoptosis, which is a key process in wound healing [57]. We also reported, to our knowledge, the first instance of metabolites found to relate to wound healing processes, such as, Isobutyl-2,2,4-trimethyl-3-hydroxypentanoate and 1,5-dimethyl-2-oxabicyclo[3.2.1]nonan-7-one to blood flow. Again, these results must be interpreted with caution until further confirmatory studies are conducted. Procrustes analysis of wound headspace metabolites identified a different set of metabolites compared to wound tissue metabolites when categorised by the same wound healing processes. This may be as a result of a different set of metabolites that are within the tissue and those that are in closer proximity to the surface, highlighting the need for both techniques to obtain a more complete representation of the wound metabolic profile.

Consistent with previous microbiome analysis, we found bacteria from *Staphylococcus*, *Corynebacterium* and *Propionibacterium* were the dominant bacterial genera contributing to the skin [58,59]. Although, the microbiome of healthy skin has been well established [60], this work highlights the predominant genera in acute wounds up to 28 days are the same as healthy skin, which may provide insight as to why acute wounds follow an expected normal healing process. Multivariate data analysis of the microbiome data showed no clear separation between the wounds at different time points in this study. This is in keeping with Oh J *et al*. who showed human skin microbial stability persists regardless of sampling time interval [61]. Therefore, acute wound microbiome stability as identified in this study may help explain why acute wounds follow an expected normal healing process. In contrast, there is no consensus regarding the predominant genera contributing to bacterial bioburden within a chronic wound [11]. There is however, evidence to suggest that the chronic wound microbiome is dynamic and faster healing is experienced in those with greater transition frequencies of the microbiome [18]. This supports the understanding that alteration of the chronic wound microbiome to that of an acute wound may be crucial in modifying the wound healing outcome.

Acute wounds at day 21 showed a statistically significant reciprocal change with the relative abundance of *Staphylococcus* decreasing and that of *Propionibacterium* increasing. This coincided with *Propionibacterium* showing a positive correlation with collagen levels in the wound. A possible hypothesis to explain this observation could be as a result of the immune system [62]. A decrease in the relative abundance of *Staphylococcus* at day 21 could be a consequence of the wound, with the initiation of an immune response in order to reduce infection risk. The coinciding rise in *Propionibacterium* could be as a result of the previously shown positive effect *Propionibacterium acnes*, through the release of coproporphyrin III, has on *Staphylococcus aureus* [63]. This hypothesis is further supported by the increase in the relative abundance of *Staphylococcus* at day 28. In contrast, *Staphylococcus* negatively correlated with collagen in the wound. The inverse relationship between *Staphylococcus* abundance and collagen synthesis is corroborated in an animal model in which a bactericidal toxin against *Staphylococcus* enhanced collagen synthesis in wounds [64]. *Propionibacterium* also negatively correlated with blood flow, which supports a previous study in a mouse breast cancer model in which *Propionibacterium* reduced angiogenesis when given in combination with melatonin [65]. We identified significant variations in the relative abundances of *Brevibacterium*, *Mycobacterium*, *Microbacterium* and *Paracoccus* across the acute wound healing time points, along with their significant relationship to wound healing processes. However, these exploratory findings warrant further study before firm conclusions can be drawn. This is because these genera have not previously been reported to play a role in acute wound healing.

This exploratory study has a number of limitations. A relatively small number of subjects were used for each part of the study, primarily due to cost implications. Therefore, the significant attribute to the variables may be limiting. However, the findings certainly justify the need for a larger scale study. The microbiome and metabolomic analyses were conducted in a different set of subjects. This was in order to have enough tissue samples to assure reliable results. This limitation was mitigated by recruiting similar subjects into each study part. The tentative identification of the compounds using the mass spectral library must be interpreted with caution as they have not been confirmed using analytical standards. In addition, part 2 of the study only recruited male participants in order to negate the effect of gender on metabolomic analyses. Future work will therefore require female participant analyses to assess for the impact of gender on skin and wound metabolomics. Further future direction could also focus on profiling the mycobiome and virome. Also, study of the wound lipid profiles and cytokines in conjunction with the biomarkers identified through this study may better help understand wound physiology.

In conclusion, this unique exploratory study successfully demonstrates the temporal and dynamic acute wound metabolome and microbiome whilst also help identifying a class of biomarkers that correspond to wound healing processes. This is encouraging and highlights the needs for further research into wound healing metabolomics. Future research is necessary to help corroborate these findings along with the need to compare the findings to the chronic wound microbiome and metabolome.

## Supporting information

S1 Fig Study design. (TIF)

S2 Fig PCA on wound headspace metabolome. PCA scores plot of PC1 *vs*. PC2 for wound headspace metabolome. The TEV of PC1 is 41.62% and for PC2 is 10.5%.
(TIF)

S3 Fig Boxplots showing the relative abundances of significantly varied genera across the examined time points. Kruskal–Wallis test with accompanying Dunn-Bonferroni post hoc analyses were performed (*n* = 5).
(TIF)

S4 Fig PCA and PC-DFA on bacterial genera. (A) PCA scores plot of PC1 *vs*. PC2. (B) PC-DFA scores plot using the first 10 PCs provided a total explained variance (TEV) of 97.4%. The numbers correspond to the subject.
(TIF)

S5 Fig PCA of part 1 non-invasive measures. (A) PCA scores plot of PC1 *vs*. PC2. The TEV of PC1 is 27.84% and for PC2 is 18.75%. (B) The corresponding PCA-loadings plot.
(TIF)

S6 Fig Boxplots showing significantly varied part 1 objective non-invasive measures across time points. Kruskal–Wallis test with accompanying Dunn-Bonferroni post hoc analyses were performed (*n* = 5).
(TIF)

S7 Fig PCA scores and PCA loadings on part 2 non-invasive measures. (A) PCA scores plot of PC1 *vs*. PC2. The TEV of PC1 is 39.87% and for PC2 is 24.39%. (B) PCA-loadings plot.
(TIF)

S8 Fig Boxplots showing significantly varied part 2 objective non-invasive measures across time points. Kruskal–Wallis test with accompanying Dunn-Bonferroni post hoc analyses were performed (*n* = 6).
(TIF)

S9 Fig Selected significant correlations between part 1 objective non-invasive measures and genera, as determined by Spearman’s rank-order correlation. (TIF)

S1 Table Study inclusion and exclusion criteria. (DOCX)

S2 Table Correlations between part 1 objective non-invasive measures and genera. (DOCX)

## Abbreviations

ASCA: analysis of variance–simultaneous component analysis
DFA: discriminant function analysis
FDR: false discovery rate
FLPI: Full-field Laser Perfusion Imaging
GC: gas chromatography
GC-MS: gas chromatography-mass spectrometry
IS: internal standard
MCC: multi capillary column
MSI: metabolomics standards initiative
NIST: national institute of standards and technology
OCT: optical coherence tomography
PC: principal component
PCA: principal component analysis
PC-DFA: principal component–discriminant function analysis
PDMS: polydimethylsiloxane
TD: thermal desorption
TEV: total explained variance
TEWL: trans-epidermal water loss
