# Supplementary figures and images for: A microbiome and metabolomic signature of phases of cutaneous healing identified by profiling sequential acute wounds of human skin: An exploratory study
Source: PLoS One. 2020 Feb 27;15(2):e0229545. doi: 10.1371/journal.pone.0229545 (PMC7046225; doi:10.1371/journal.pone.0229545)

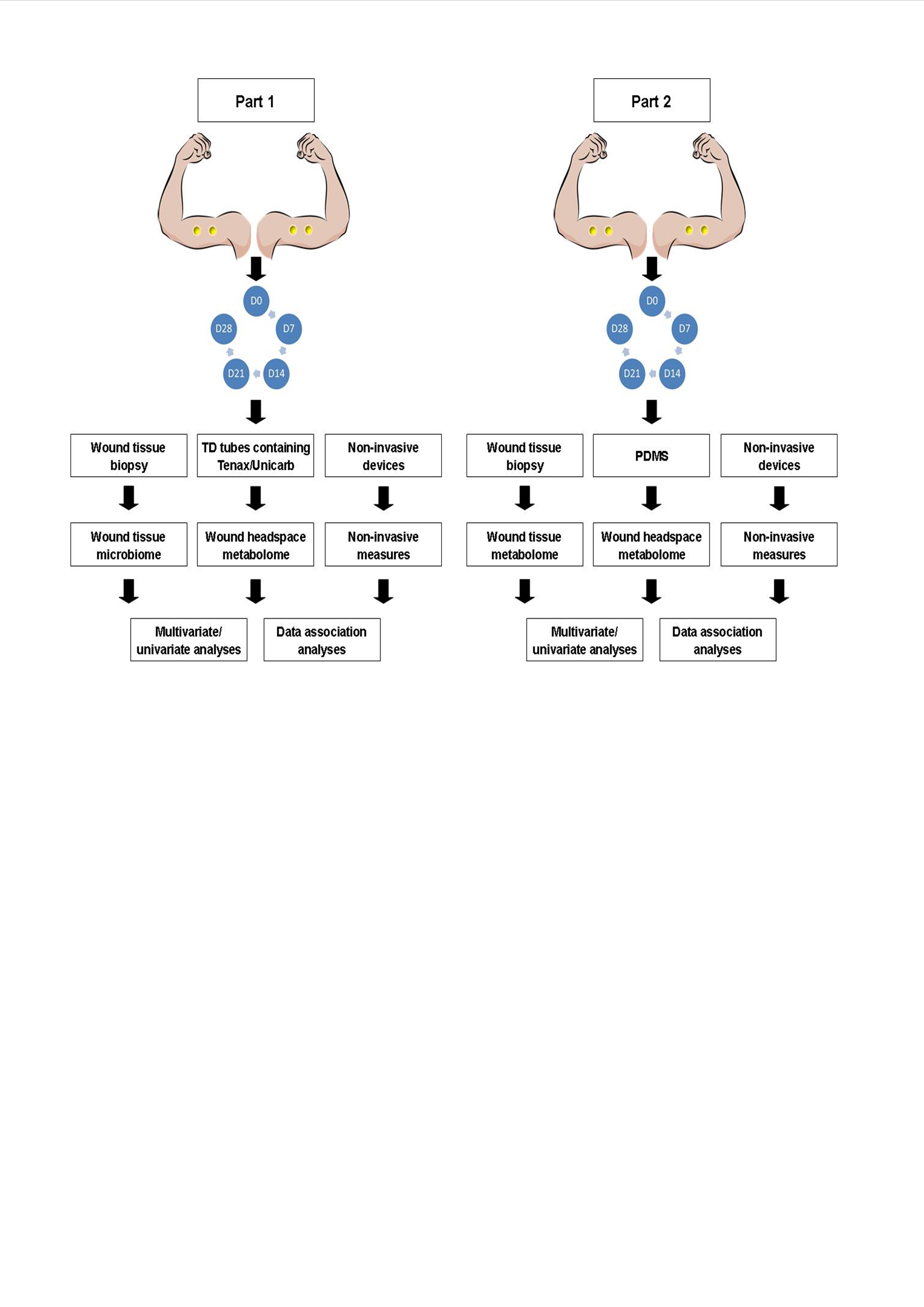

Supplement: S1 Fig — (TIF) [file pone.0229545.s001.tif]

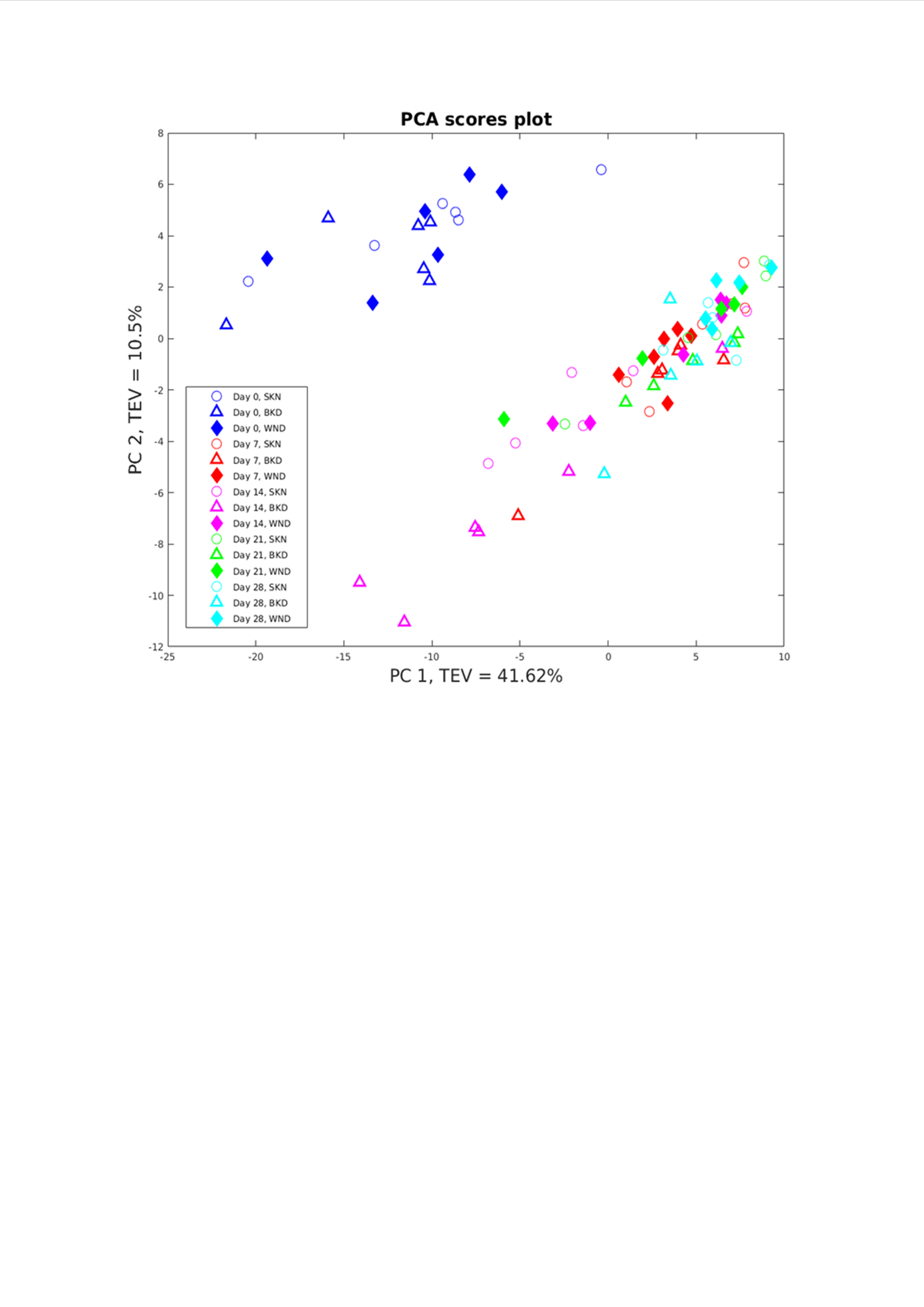

Supplement: S2 Fig — PCA scores plot of PC1 vs. PC2 for wound headspace metabolome. The TEV of PC1 is 41.62% and for PC2 is 10.5%. (TIF) [file pone.0229545.s002.tif]

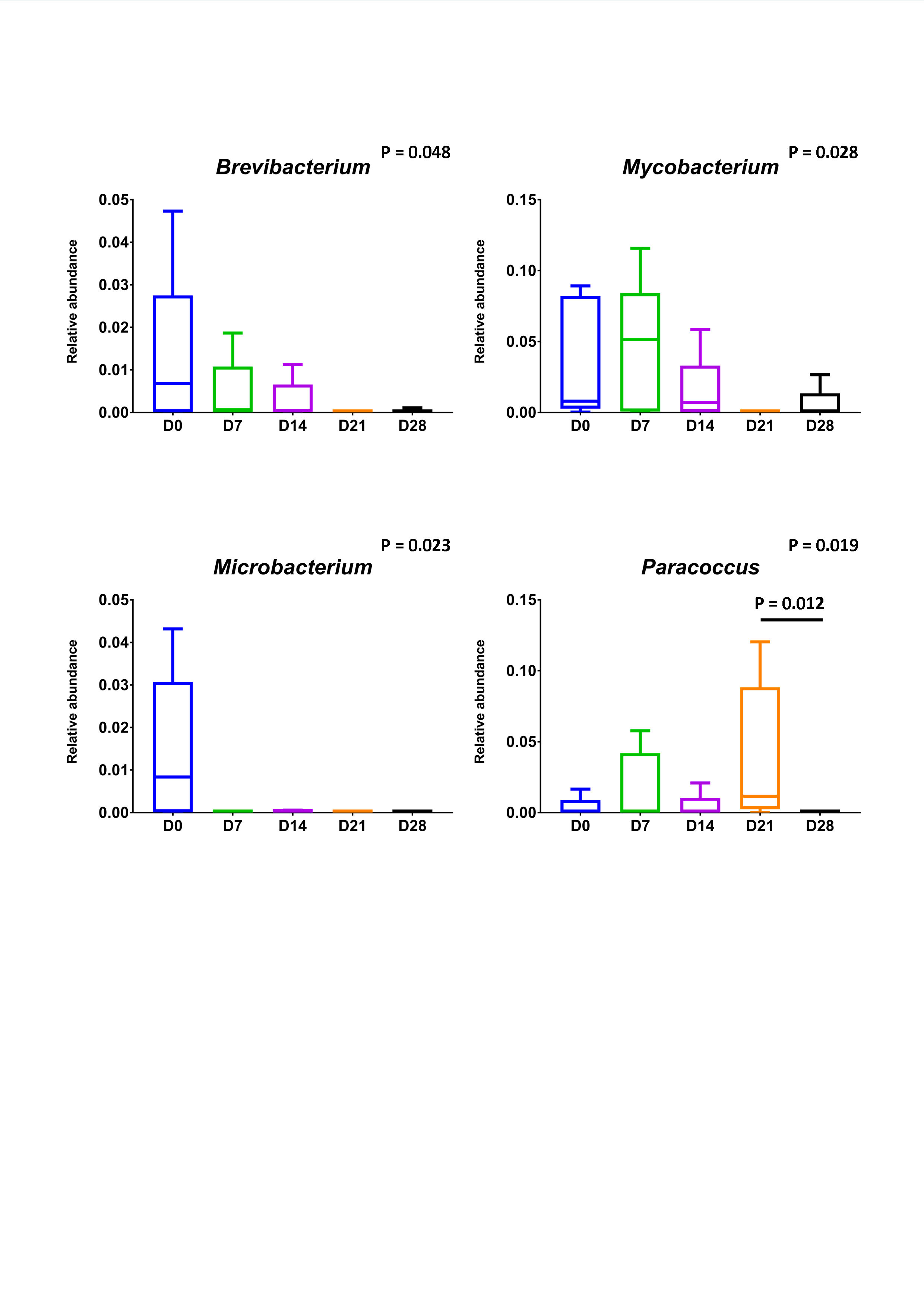

Supplement: S3 Fig — Kruskal–Wallis test with accompanying Dunn-Bonferroni post hoc analyses were performed (n = 5). (TIF) [file pone.0229545.s003.tif]

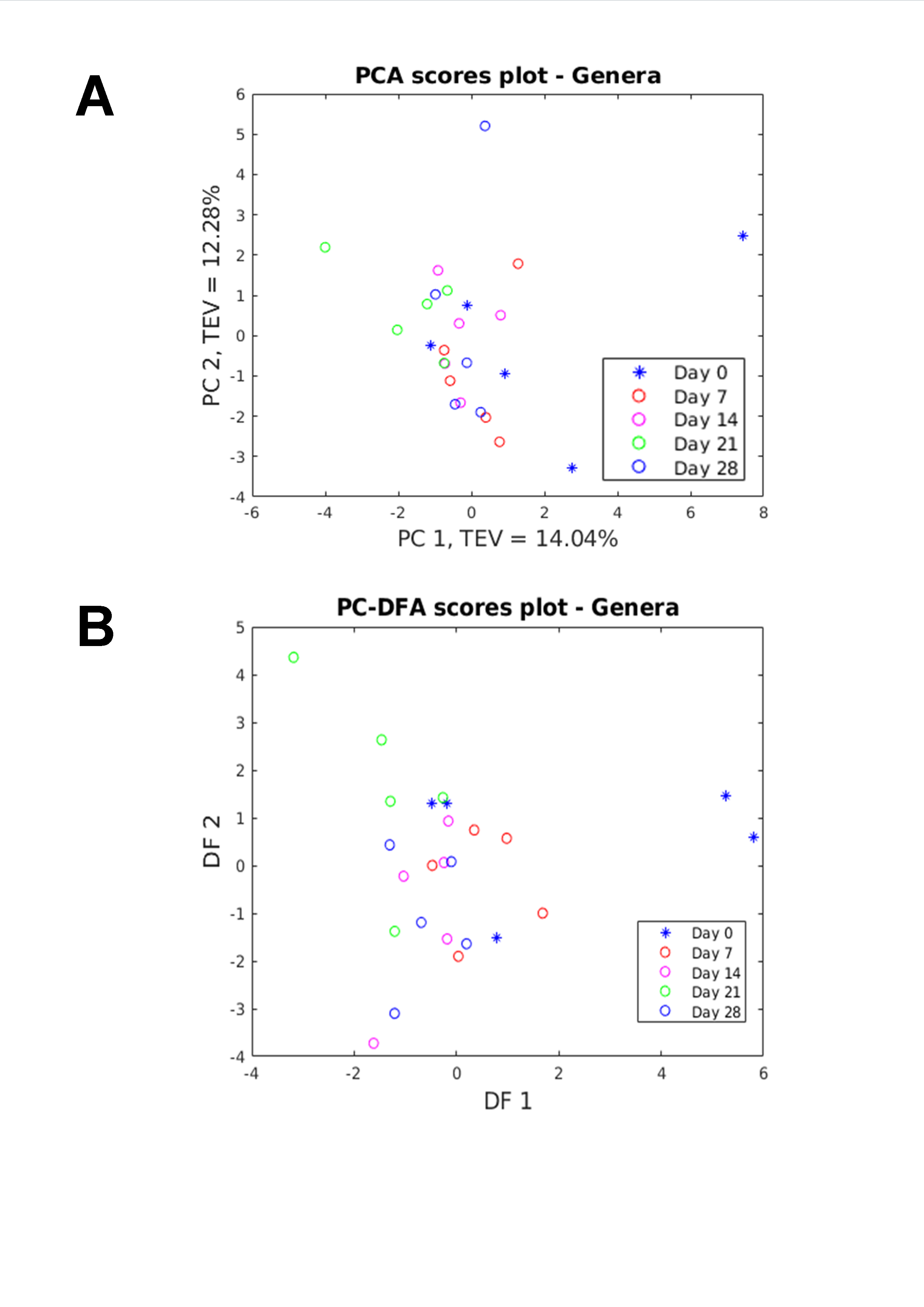

Supplement: S4 Fig — (A) PCA scores plot of PC1 vs. PC2. (B) PC-DFA scores plot using the first 10 PCs provided a total explained variance (TEV) of 97.4%. The numbers correspond to the subject. (TIF) [file pone.0229545.s004.tif]

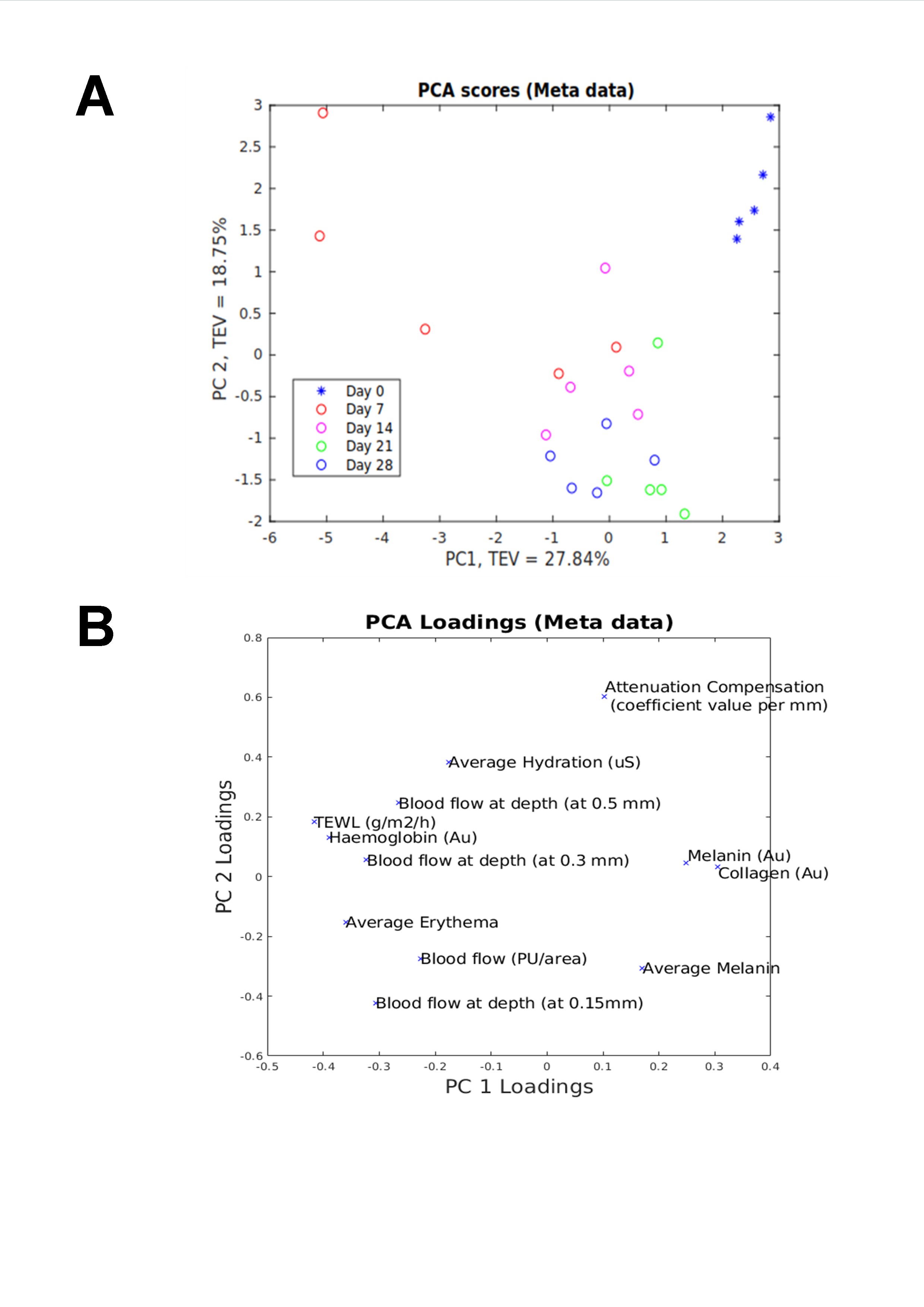

Supplement: S5 Fig — (A) PCA scores plot of PC1 vs. PC2. The TEV of PC1 is 27.84% and for PC2 is 18.75%. (B) The corresponding PCA-loadings plot. (TIF) [file pone.0229545.s005.tif]

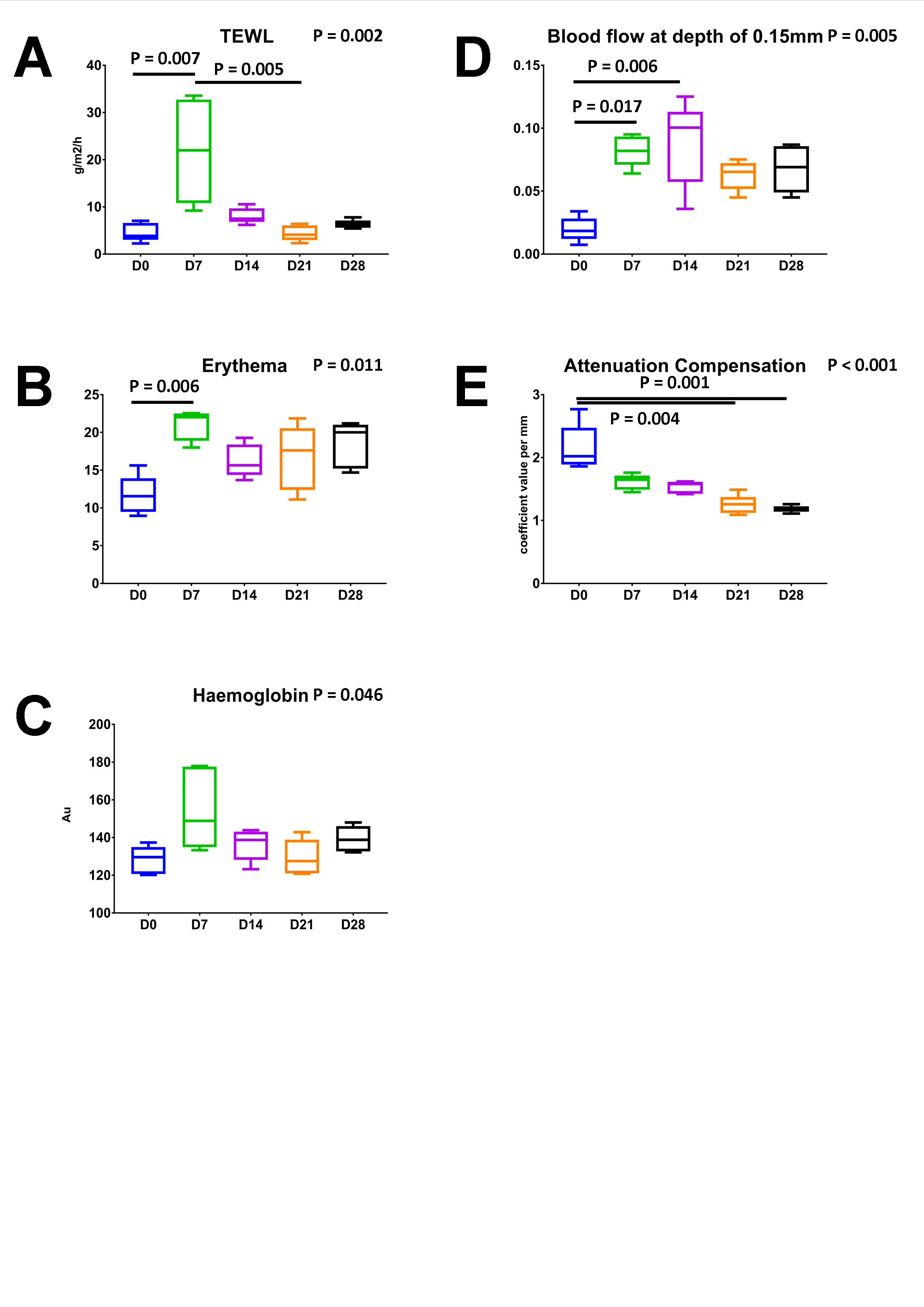

Supplement: S6 Fig — Kruskal–Wallis test with accompanying Dunn-Bonferroni post hoc analyses were performed (n = 5). (TIF) [file pone.0229545.s006.tif]

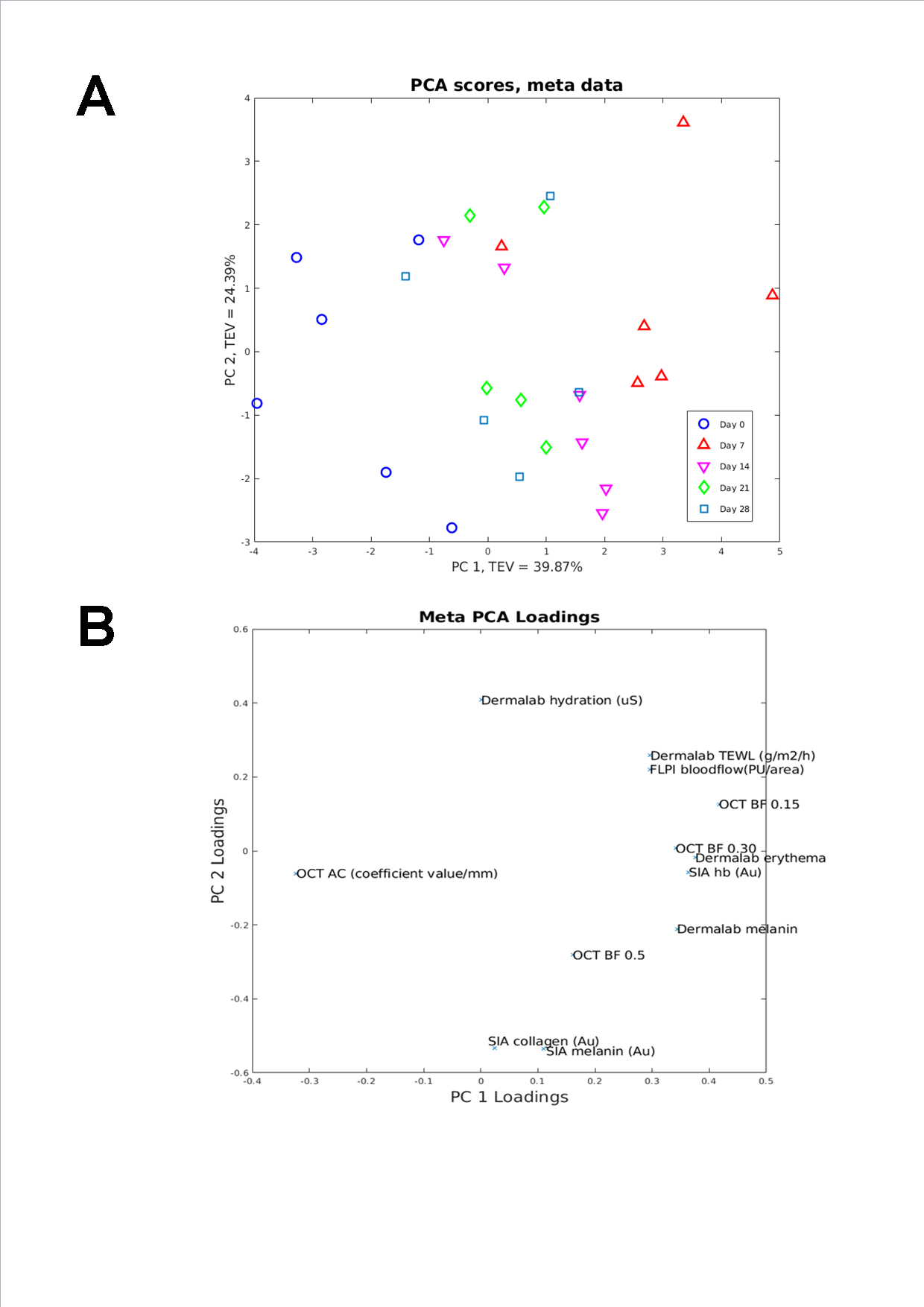

Supplement: S7 Fig — (A) PCA scores plot of PC1 vs. PC2. The TEV of PC1 is 39.87% and for PC2 is 24.39%. (B) PCA-loadings plot. (TIF) [file pone.0229545.s007.tif]

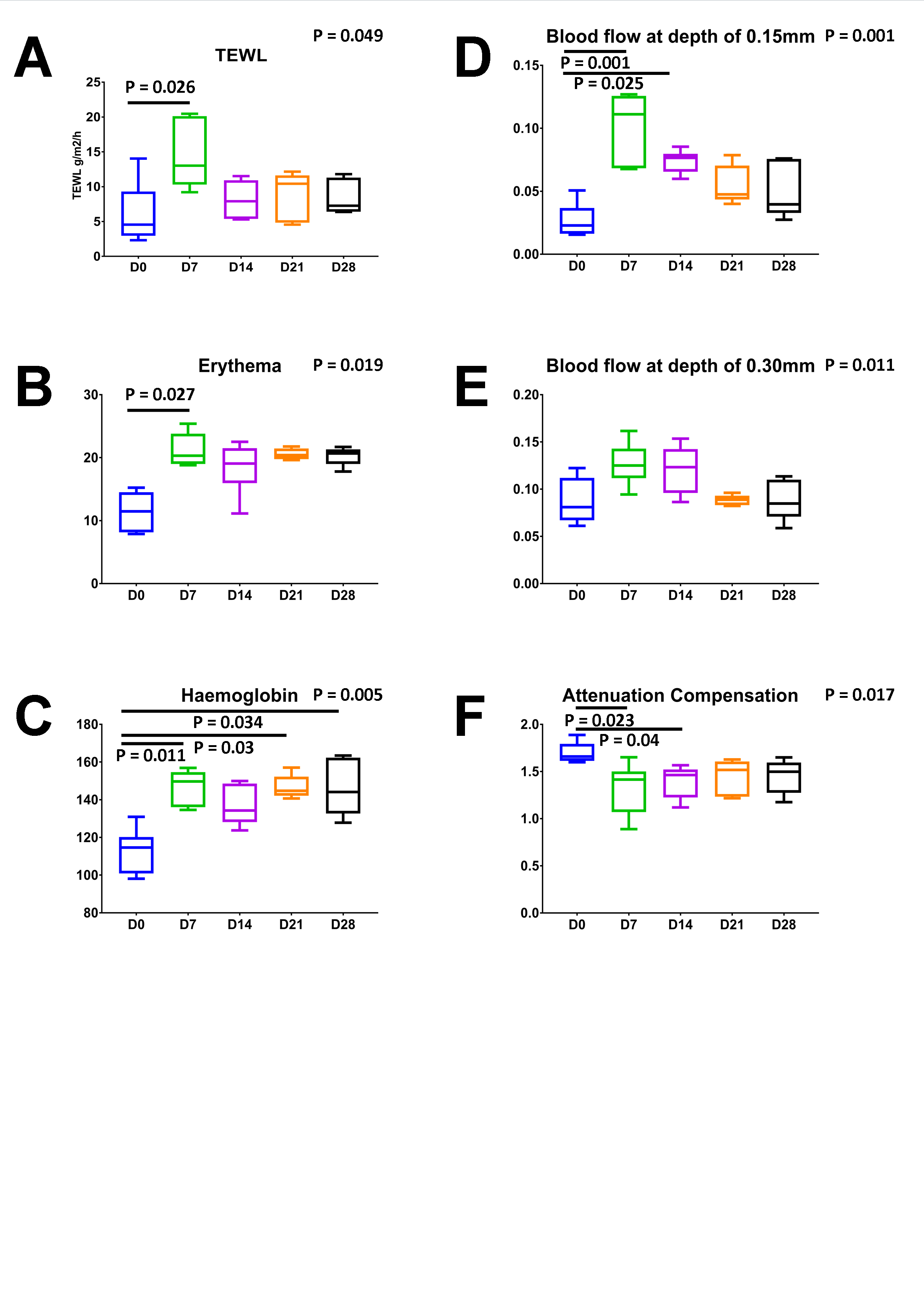

Supplement: S8 Fig — Kruskal–Wallis test with accompanying Dunn-Bonferroni post hoc analyses were performed (n = 6). (TIF) [file pone.0229545.s008.tif]

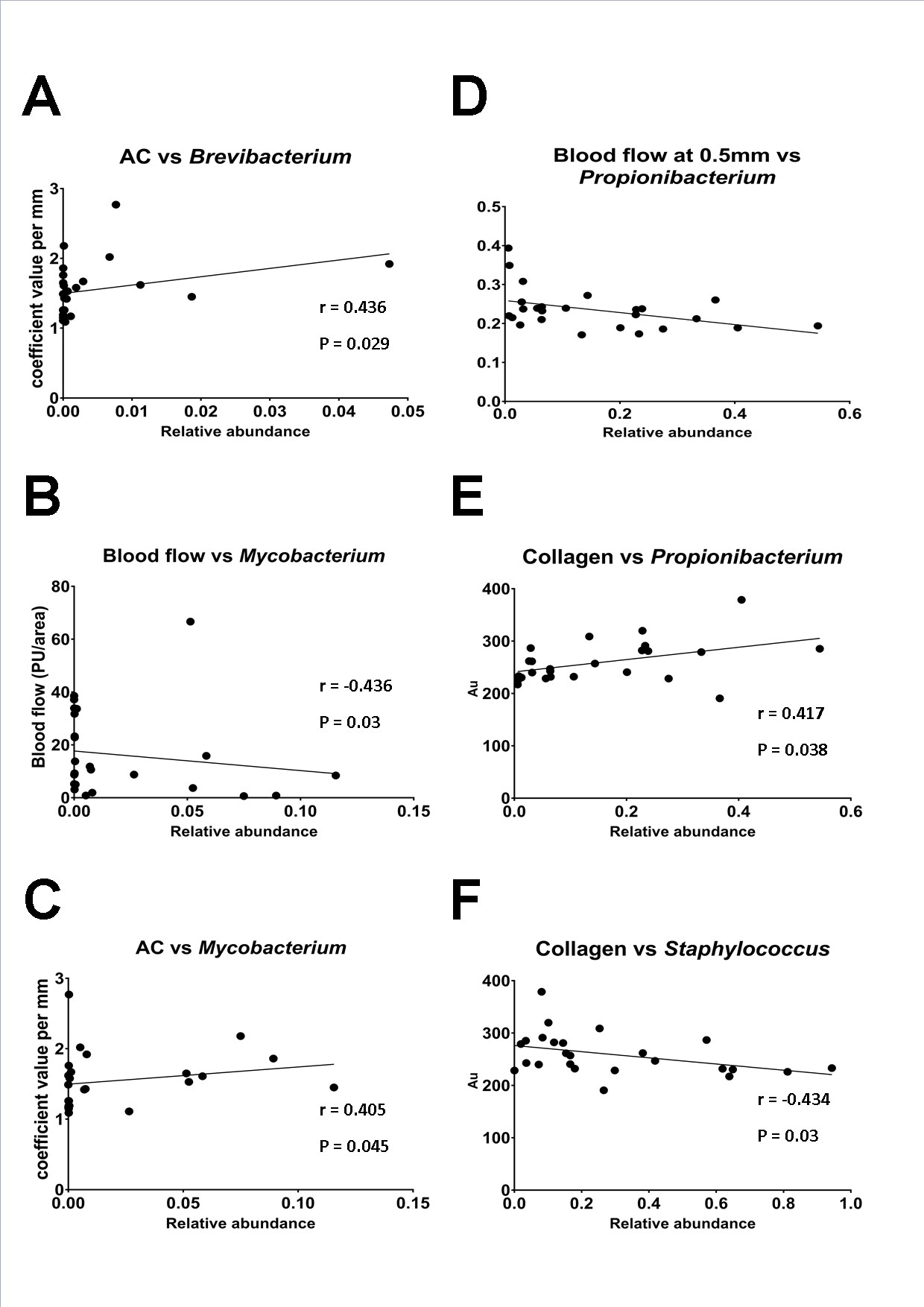

Supplement: S9 Fig — (TIF) [file pone.0229545.s009.tif]
