# Supplementary material for: A microbiome and metabolomic signature of phases of cutaneous healing identified by profiling sequential acute wounds of human skin: An exploratory study
Source: PLoS One. 2020 Feb 27;15(2):e0229545. doi: 10.1371/journal.pone.0229545 (PMC7046225; doi:10.1371/journal.pone.0229545)
Supplement: S1 Table — (DOCX) [file pone.0229545.s010.docx]

**S1 Table. Study inclusion and exclusion criteria**

| **Inclusion Criteria** |
| --- |
| - Male or Female - Aged 16 or over - Able to understand study requirements - Able to attend all follow-up visits - Able to provide written consent if competent - Weight between 40-150 kg with a body mass index of 20-45 kg/m^2^. |
|  |
| **Exclusion Criteria** |
|  |
| - Subjects who do not give consent or withdraw consent to take part in the study - Aged less than 16 years - Subjects with an active skin disorder considered to adversely affect the healing of the acute wound by the investigator - Subjects who have a history or evidence of keloid scarring or previous scarring on the medial aspect of the upper arms (self-reported or determined by physical examination) - Subjects who are pregnant or are planning to conceive in the next 3 months - Subjects with any likely wound healing impairment due to a clinically significant medical condition such as renal, hepatic, haematological, neurological or immune disease - Malignancy – diagnosed or treated within the last 5 years - Immunosuppressive, radiation or chemotherapy within the last 3 months - Current anticoagulant therapy (e.g warfarin), systemic steroids, hormone replacement therapy or any investigational drugs taken in the previous month - Subjects with evidence of illicit drug abuse - Subjects who are known to have hepatitis B or C infection including hepatitis B surface antigen, hepatitis B core antibodies or hepatitis C antibodies - Subjects who are HIV positive - Any subject who in the opinion of the investigator is unable to fully understand the requirements of the trial, consent or who is unable to return for follow-up appointments - Any subject who becomes systemically unwell during the research process due to external study causes - Subjects involved in other studies in the past 2 months prior to day 0 |
