# Supplementary material for: A microbiome and metabolomic signature of phases of cutaneous healing identified by profiling sequential acute wounds of human skin: An exploratory study
Source: PLoS One. 2020 Feb 27;15(2):e0229545. doi: 10.1371/journal.pone.0229545 (PMC7046225; doi:10.1371/journal.pone.0229545)
Supplement: S2 Table — (DOCX) [file pone.0229545.s011.docx]

**S2 Table. Correlations between part 1 objective non-invasive measures and genera**

| **Non-invasive modality** | **Genera** | ***r*** | ***P* value** |
| --- | --- | --- | --- |
| **TEWL (g/m^2^/h)** | *Corynebacterium* | -0.458 | 0.021 |
| **Average Hydration (µS)** | *Phyllobacterium* | -0.415 | 0.039 |
| **Average Melanin** | *Prevotella* | 0.428 | 0.037 |
|  | *Sphingomonas* | 0.44 | 0.031 |
|  | *Acinetobacter* | -0.417 | 0.042 |
| **Blood flow (PU/area)** | *Corynebacterium* | -0.397 | 0.049 |
|  | *Gordonia* | -0.505 | 0.01 |
|  | *Mycobacterium* | -0.435 | 0.03 |
| **Melanin (Au)** | *Corynebacterium* | 0.63 | 0.001 |
|  | *Anaerococcus* | 0.483 | 0.015 |
|  | *Pseudomonas* | 0.465 | 0.019 |
| **Collagen (Au)** | *Corynebacterium* | 0.428 | 0.033 |
|  | *Propionibacterium* | 0.417 | 0.038 |
|  | *Staphylococcus* | -0.434 | 0.03 |
| **Blood flow at depth (at 0.3 mm)** | *Lactobacillus* | 0.399 | 0.048 |
|  | *Sphingomonas* | 0.677 | <0.001 |
|  | *Acinetobacter* | -0.629 | 0.001 |
| **Blood flow at depth (at 0.5 mm)** | *Propionibacterium* | -0.438 | 0.028 |
|  | *Lactobacillus* | 0.471 | 0.017 |
|  | *Sphingomonas* | 0.763 | <0.001 |
|  | *Acinetobacter* | -0.635 | 0.001 |
| **Attenuation Compensation (coefficient value per mm)** | *Brevibacterium* | 0.436 | 0.029 |
|  | *Mycobacterium* | 0.405 | 0.045 |
|  | *Phenylobacterium* | -0.465 | 0.019 |
|  | *Sphingomonas* | 0.429 | 0.032 |
